# The genetic variability and evolution of red-spotted grouper nervous necrosis virus quasispecies can be associated with its virulence

**DOI:** 10.3389/fmicb.2023.1182695

**Published:** 2023-06-15

**Authors:** Sergio Ortega-del Campo, Luis Díaz-Martínez, Patricia Moreno, Esther García-Rosado, M. Carmen Alonso, Julia Béjar, Ana Grande-Pérez

**Affiliations:** ^1^Departamento de Biología Celular, Genética y Fisiología, Facultad de Ciencias, Universidad de Málaga, Málaga, Spain; ^2^Centro de Supercomputación y Bioinnovación (SCBI), Universidad de Málaga, Málaga, Spain; ^3^Departamento de Microbiología, Facultad de Ciencias, Universidad de Málaga, Málaga, Spain; ^4^Instituto de Biotecnología y Desarrollo Azul, IBYDA, Universidad de Málaga, Málaga, Spain; ^5^Instituto de Hortofruticultura Subtropical y Mediterránea “La Mayora”, Universidad de Málaga- Consejo Superior de Investigaciones Científicas (IHSM-UMA-CSIC), Málaga, Spain

**Keywords:** NNV, virulence, quasispecies, NGS, genetic variability, haplotype reconstruction, recombination, comparative analysis

## Abstract

Nervous necrosis virus, NNV, is a neurotropic virus that causes viral nervous necrosis disease in a wide range of fish species, including European sea bass (*Dicentrarchus labrax*). NNV has a bisegmented (+) ssRNA genome consisting of RNA1, which encodes the RNA polymerase, and RNA2, encoding the capsid protein. The most prevalent NNV species in sea bass is red-spotted grouper nervous necrosis virus (RGNNV), causing high mortality in larvae and juveniles. Reverse genetics studies have associated amino acid 270 of the RGNNV capsid protein with RGNNV virulence in sea bass. NNV infection generates quasispecies and reassortants able to adapt to various selective pressures, such as host immune response or switching between host species. To better understand the variability of RGNNV populations and their association with RGNNV virulence, sea bass specimens were infected with two RGNNV recombinant viruses, a wild-type, rDl956, highly virulent to sea bass, and a single-mutant virus, Mut270Dl965, less virulent to this host. Both viral genome segments were quantified in brain by RT-qPCR, and genetic variability of whole-genome quasispecies was studied by Next Generation Sequencing (NGS). Copies of RNA1 and RNA2 in brains of fish infected with the low virulent virus were 1,000-fold lower than those in brains of fish infected with the virulent virus. In addition, differences between the two experimental groups in the Ts/Tv ratio, recombination frequency and genetic heterogeneity of the mutant spectra in the RNA2 segment were found. These results show that the entire quasispecies of a bisegmented RNA virus changes as a consequence of a single point mutation in the consensus sequence of one of its segments. Sea bream (*Sparus aurata*) is an asymptomatic carrier for RGNNV, thus rDl965 is considered a low-virulence isolate in this species. To assess whether the quasispecies characteristics of rDl965 were conserved in another host showing different susceptibility, juvenile sea bream were infected with rDl965 and analyzed as above described. Interestingly, both viral load and genetic variability of rDl965 in seabream were similar to those of Mut270Dl965 in sea bass. This result suggests that the genetic variability and evolution of RGNNV mutant spectra may be associated with its virulence.

## 1. Introduction

Aquaculture is an increasingly important activity, with a major global economic impact. The main constraint to aquaculture development is the occurrence of infectious disease outbreaks. Furthermore, the rapid growth of aquaculture, intensive farming techniques, introduction of new species and the environmental change provide ideal conditions for the emergence of new pathogens (Pérez-Sánchez et al., 2018).

Nervous necrosis virus (NNV), belonging to the *Betanodavirus* genus (*Nodaviridae* family), is one of the most harmful pathogens to the aquaculture industry, causing viral nervous necrosis (VNN), also known as viral encephalopathy and retinopathy (VER), a severe disease affecting the central nervous system (CNS) of numerous marine and freshwater fish species worldwide (Thiéry et al., 2004; Kara et al., 2014; Doan et al., 2017; Bandín and Souto, 2020). VER particularly affects hatchery-reared larvae and juveniles, causing high mortality rate (80–100%) (Le Breton et al., 1997; Munday et al., 2002; Volpe et al., 2020).

NNV genome consists of two single-stranded RNA molecules of positive polarity (+ ssRNA), RNA1 and RNA2. RNA1 (3.1 kb) contains a single open reading frame (ORF) that encodes an RNA-dependent RNA polymerase (RdRp), also known as protein A, whereas RNA2 (1.4 kb) encodes the capsid protein (Cp) (Munday et al., 2002; Chen et al., 2015; Bandín and Souto, 2020). Replication of the viral genome involves a subgenomic RNA (sgRNA3, 371–378 nt), transcribed from the 3′ end of the RNA1 segment, that encodes two non-structural proteins, B1 and B2 (Iwamoto et al., 2001; Bandín and Souto, 2020).

There are four NNV species based on RNA2 sequencing: barfin flounder nervous necrosis virus (BFNNV), red-spotted grouper nervous necrosis virus (RGNNV), striped jack nervous necrosis virus (SJNNV), and tiger puffer nervous necrosis virus (TPNNV) (Nishizawa et al., 1997). The main NNV reported in the Mediterranean area are RGNNV and SJNNV, which affect European sea bass (*Dicentrarchus labrax*) and sea bream (*Sparus aurata*). Moreover, RGNNV-SJNNV reassortant isolates, presenting both segment combinations, SJNNV/RGNNV and RGNNV/SJNNV (RNA1/RNA2), have been isolated from several fish species (Toffolo et al., 2007; Olveira et al., 2009; Toffan et al., 2017; Qin et al., 2020; Volpe et al., 2020). Specifically, European sea bass is highly susceptible to RGNNV infections, whereas sea bream is resistant to RGNNV but susceptible to SJNNV and RGNNV/SJNNV infections (Castric et al., 2001; Olveira et al., 2009; Chaves-Pozo et al., 2012; Vendramin et al., 2014; Souto et al., 2015b; Carballo et al., 2016; Toffan et al., 2017; Volpe et al., 2020; Pereiro et al., 2023).

The fitness of NNV in its hosts is determined by a high genetic diversity, with reassortment as one of the main sources of genetic diversity (Panzarin et al., 2016). Furthermore, reassortment has been associated with colonization of new fish species. Several studies have shown that both sea bream and sea bass are susceptible to RGNNV/SJNNV, although it has been suggested that sea bass could act as a reservoir, since a high viral load has been detected in asymptomatic specimens (Biasini et al., 2022). As RNA viruses, NNV possess high mutation rates that, along with their small genomic size, result in mutant spectra characterized by high variability and genetic heterogeneity (Sugaya et al., 2009; Panzarin et al., 2012). Although NNV genetic variation has been poorly examined, several studies have shown that NNV is organized as quasispecies (Hick et al., 2010; Chen et al., 2020).

**Figure 1:**
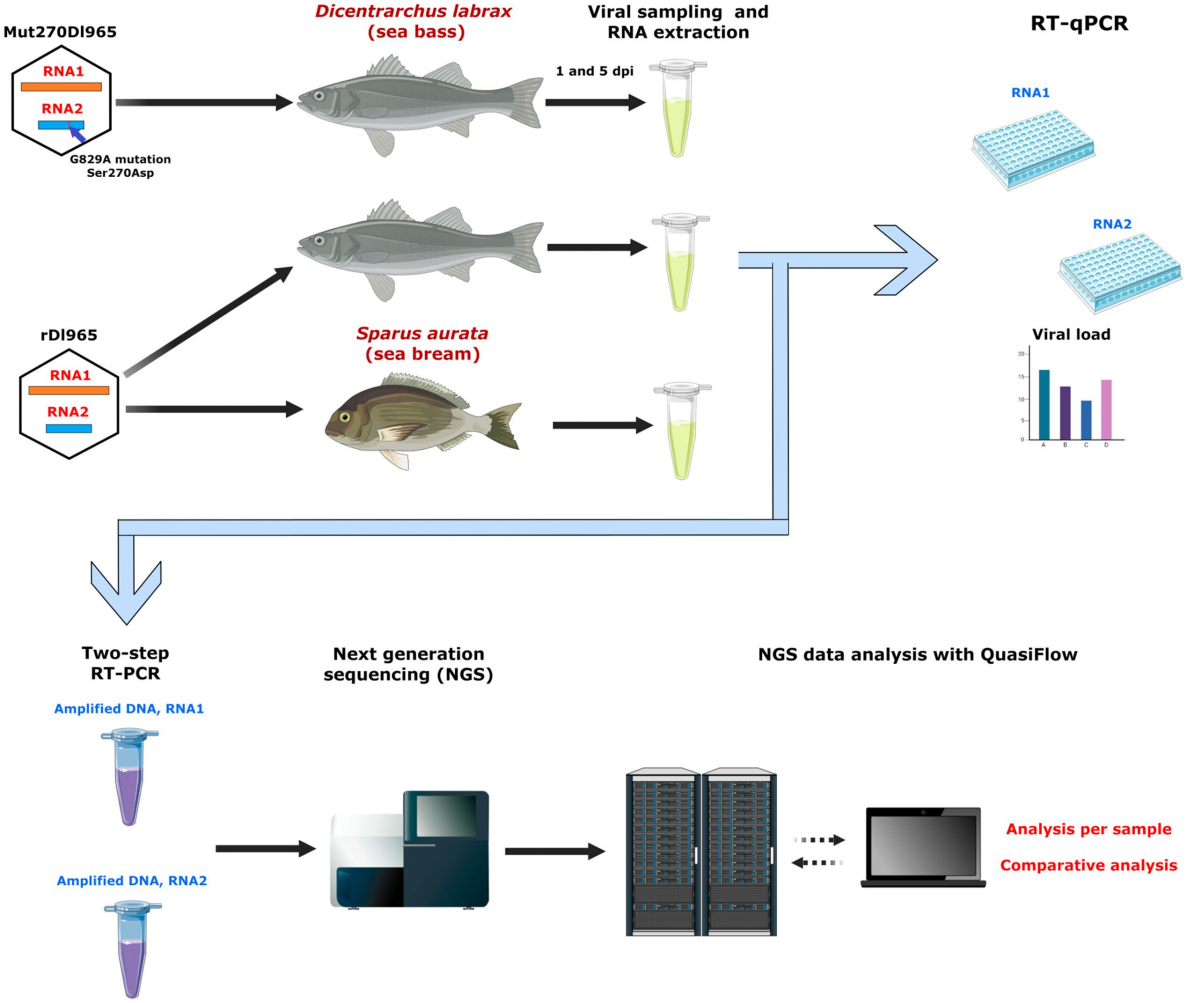
Outline of the study experimental design. Sea bass specimens were inoculated with the recombinant RGNNV viruses rDl965 (GenBank accession number NC_008040 for RNA1 sequence and NC_008041 for RNA2 sequence) and Mut270Dl965 (harboring the G829A mutation in RNA2 that causes the Ser270Asp amino acid change in the capsid protein). Sea bream were also inoculated with rDl965. Brain samples were collected at 1 and 5 days post infection (dpi). Viral genome was quantified for each RNA segment by RT-qPCR, and the whole segments were amplified by two-step RT-PCR, followed by NGS sequencing and analysis of mutant spectra using QuasiFlow software.

The role of quasispecies in RGNNV virulence *in vivo* has been analyzed in the present study (experimental design shown in Figure 1). To that aim, two viruses that had been obtained by reverse genetics in a previous study were used, namely a recombinant RGNNV (rDl965), causing 73.3% mortality in sea bass, and an attenuated RGNNV (Mut270Dl965), with 20% mortality in sea bass (Moreno et al., 2019). Mut270Dl965 had a non-synonymous mutation at position 270 of the Cp protein, which produces a serine to asparagine amino acid change. Whole genome mutant spectra were analyzed by New Generation Sequencing (NGS), genetic variability and diversity were estimated, haplotypes composing the mutant spectra of both genomic segments were reconstructed, and a statistical comparison of all samples was performed. According to our results, the presence of the non-synonymous mutation at position 270 of the Cp protein, which is a virulence determinant of RGNNV in sea bass, leads to a change in the genetic quasispecies variability. To evaluate whether the quasispecies characteristics of rDl965, highly virulent to sea bass, were conserved in a different host showing low susceptibility, sea bream juveniles were also infected with rDl965. Interestingly, results showed that rDl965 quasispecies in sea bream are similar to Mut270Dl965 quasispecies in sea bass.

## 2. Materials and methods

### 2.1. Virus and cell culture

The recombinant rDl965 virus had been generated by reverse genetics from the RGNNV isolate SpDl_IAusc965.09, obtained from a diseased European sea bass (Moreno et al., 2019). Mut270Dl965, derived from rDl965, carries a site-specific mutation at position 830 within the RNA2 segment (aa270 of the Cp sequence, Ser270Asp) (Moreno et al., 2019). Both recombinant viruses were propagated using the E11 cell line (Iwamoto et al., 2001), at 25°C, with Leibovitz L-15 medium (Gibco) supplemented with 2% foetal bovine serum (FBS, Gibco), 100 units/mL penicillin and 10 mg/mL streptomycin (Sigma) until complete cytopathic effects (CPE) were observed. The resulting viral suspension was titrated following the 50% tissue culture infective dose method (TCID_50_) (Reed and Muench, 1938) and stored at −80°C until used.

### 2.2. European sea bass and sea bream challenge

Juvenile sea bass (8 g, average weight) were distributed into 3 experimental groups (*n* = 20): (i) rDl965-infected group, (ii) Mut270Dl965-infected group, and (iii) L-15-injected group, as negative control. Specimens were infected intramuscularly at a dose of 2 × 10^6^ TCID_50_/fish. Juvenile sea bream specimens (8 g, average weight) were only injected with rDl965 (2 × 10^6^ TCID_50_/fish). At 1 and 5 days post-injection (dpi), three animals per group were sacrificed by MS-22 overdose, and brains were individually collected and stored in liquid nitrogen for subsequent analyses (Table 1).

**Table 1:** Samples analyzed in this study, organized by host species, days post-infection (dpi), and viruses inoculated.

Virus	dpi	Host	
		*Dicentrarchus labrax*	*Sparus aurata*
rDl965	1	Dla_WT_1_r1	Sau_WT_1_r1
		Dla_WT_1_r2	Sau_WT_1_r2
		Dla_WT_1_r3	Sau_WT_1_r3
	5	Dla_WT_5_r1	Sau_WT_5_r1
		Dla_WT_5_r2	Sau_WT_5_r2
		Dla_WT_5_r3	Sau_WT_5_r3
Mut270Dl965	1	Dla_Mut_1_r1	
		Dla_Mut_1_r2	
		Dla_Mut_1_r3	
	5	Dla_Mut_5_r1	
		Dla_Mut_5_r2	
		Dla_Mut_5_r3	

### 2.3. Ethics statement

Fish were always handled according to the European Union guidelines for the handling of laboratory animals (Directive 2010/63/UE), applying the lowest stress-generating conditions of light, oxygen and feeding. To minimize fish suffering, trials were accomplished in accordance with the Bioethics Committee of Junta de Andalucia (number: 09/08/2019/136).

### 2.4. RNA extraction, amplification, and next-generation sequencing

Total RNA was extracted from brain using TRI reagent solution, quantified using the NanoDrop ND 1000 microdrop spectrophotometer system (Thermo Fisher), and diluted to 800 ng/μl. cDNA synthesis was performed in duplicate using AMV RT (Promega), with 1 mM dNTP, 0.4 μM reverse primer (3’R1_965 for RNA1 and 3’R2_965 for RNA2), 4 u/μl RNasin Ribonuclease Inhibitor, 352 ng/μl RNA and 30 u AMV RT. The maximum volume of RNA was added to the reaction to avoid bottlenecks and loss of low-frequency haplotypes of the viral quasispecies. After incubation at 42°C for 60 min, duplicate RT reactions were mixed and cDNA was quantified. Duplicate PCR reactions for each genomic segment were performed using 0.25 mM dNTP, 0.2 μM specific primers (T7_5’R1_965/3’R1_965 for RNA1, and T7_5’R2_965/3’R2_965 for RNA2) (Supplementary material S1), 4–8 ng of cDNA and 40 u *PfuUltra* II Fusion HS DNA polymerase (Agilent). Amplification reaction was carried out according to the manufacturer’s instructions, including an initial denaturation cycle of 1 min at 95°C, followed by 30 cycles of 20 s at 95°C for denaturation, 20 s at 55°C for annealing and 2 min at 72°C for elongation. A final elongation cycle at 72°C for 3 min was included as a final step. As a result, two amplicons were obtained, corresponding to RNA1 and RNA2 viral segments. Duplicate PCR products of each genomic segment per sample were pooled prior to sequencing.

DNA library was prepared using the Nextera XT DNA Library Prep Kit (Illumina), according to the manufacturer’s protocol. Briefly, PCR products were subjected to tagmentation, which enzymatically cleaved and tagged DNA with Illumina adapters. DNA was then subjected to a 12-cycle PCR, for indexing/barcoding, with a unique combination of 15 and 17 index primers. Amplification products were purified using Agencourt AMPure XP beads (Beckman Coulter Inc., CA, USA) and manually normalized. High-throughput sequencing was performed with Nextseq 550 Reagent Mid Output Kit v2.5 (300-cycle, Illumina) to generate 3–10 million 150-nucleotide paired-end reads per sequencing run, using the proprietary Illumina Nextseq550 System. DNA library preparation and sequencing were performed at the Supercomputing and Bioinnovation Center (SCBI) of the University of Malaga (Spain).

### 2.5. Viral genome quantification

**Figure 2:**
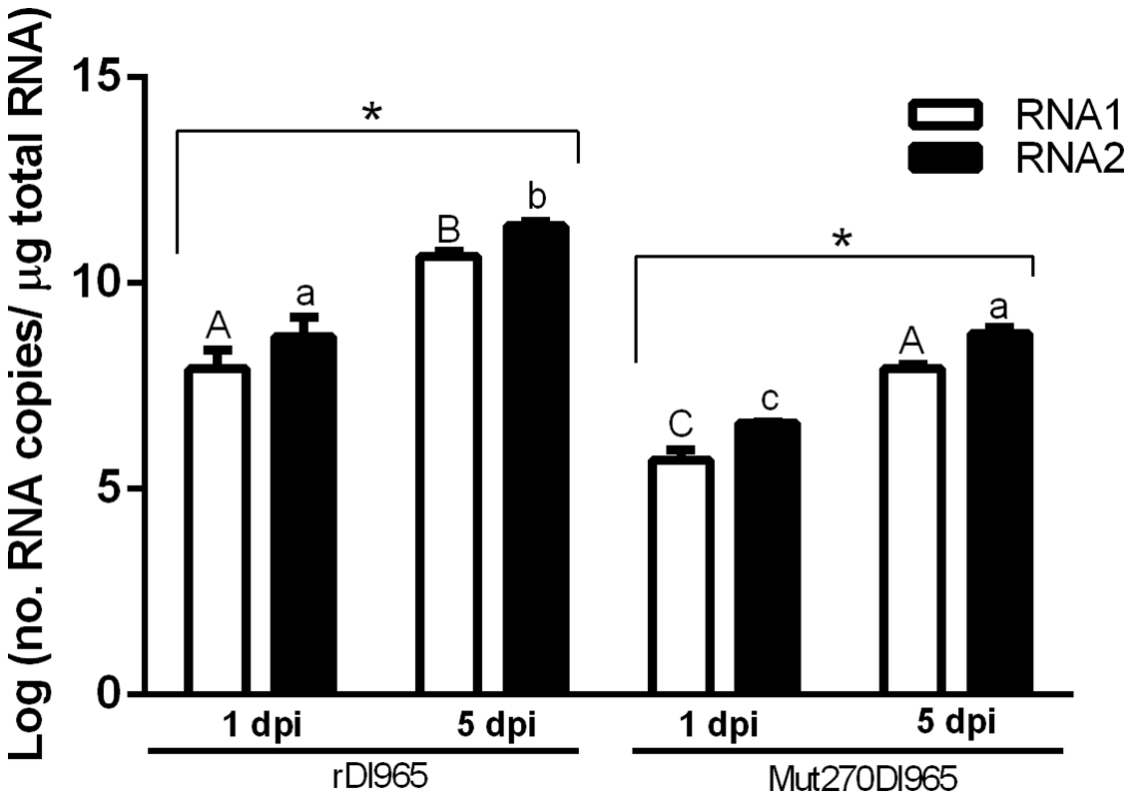
Number of RNA1 and RNA2 molecules per μg of total RNA in brain of European sea bass infected with rDl965 or Mut270Dl965. Student’s t-test and one-way ANOVA were used to compare values between hosts and post-infection times. Values of *p* < 0.05 were considered significant. Results are mean ± SD (*n* = 3). Different uppercase and lowercase letters indicate significant differences in RNA1 or RNA2 copy numbers, respectively, between experimental groups. Asterisks denote significant differences between sample times within experimental groups.

RNA1 and RNA2 quantification was performed by absolute RT-qPCR following the procedure described by Moreno et al. (2016, 2019). Specific pairs of primers for RNA1 and RNA2 amplification were RG-RNA1-F/RG-RNA1-R (Lopez-Jimena et al., 2011), and RG_965_RNA2 F4/RG_965_RNA2 R1 (Moreno et al., 2016), respectively. RNA was purified from rDl965 (10^7^ TCID_50_/ml), quantified by DS-11 Fx + (Denovix), and diluted to be used as a reference standard curve. RNA was reverse transcribed using AMV reverse transcriptase (Promega). Significant differences in viral load between sea bass samples infected with rDl965 or Mut270Dl965, as well as between sea bass and sea bream samples infected with rDl965, were tested by Student’s t-tests and one-way ANOVA. Statistical analyses were performed using GraphPad software (Figure 2; Figure 5).

### 2.6. Genetic variability of quasispecies

The genetic complexity and heterogeneity of RGNNV-mutant spectra were analyzed using QuasiFlow, an open-source workflow for the analysis of viral quasispecies using NGS data (Seoane et al., 2022). QuasiFlow was run on PICASSO supercomputer (SCBI, University of Malaga, Spain). QuasiFlow analyses were performed separately for each viral segment (RNA1 and RNA2). The analyses were performed using the default conditions.

The composition and distribution of mutations (SNPs, InDels) was firstly obtained. We analyzed possible biases in the types of substitutions per group of samples, classified by time of sampling, virus and host. The expected frequency for a given substitution (substitution of nt X by nt Y, f E X → Y) was calculated assuming that all substitutions were equally likely following this formula:

$$fE\;X\to Y=\frac{Px*M}{3}$$

P_X_ = fractional proportion of nucleotides X (= A, G, T or C) in parental sequence.

M = total mutations observed.

Significant deviations of the expected number of each substitution were tested using a 2 × 2 chi-square test.

Mutation frequency was calculated as the proportion of mutated residues in the alignment compared to the reference sequence (Seoane et al., 2022). Nucleotide diversity, measured as the average number of nucleotide differences between any two genomes of the quasispecies (Gregori et al., 2016), was also estimated. Haplotypes were reconstructed and quasispecies heterogeneity was analyzed using the normalized Shannon index, i.e., a measure of diversity based on haplotype frequencies (Gregori et al., 2016). The workflow established an iterative analysis of the reads, joining them if they are identical within 90% of their length creating haplotypes. In this way, the reads cycle by cycle decreased in number, and increased in nucleotide length until all possible haplotypes were generated. Once each haplotype was reconstructed, and the variations of each mutant spectrum were identified, the viral quasispecies were represented by networks connecting haplotypes with their mutations (SNPs and InDels) (Seoane et al., 2022).

Recombination events, as well as breakpoints in genome segments, were detected for each sample. Recombination frequency, that is, the number of recombinant reads divided by the total number of reads analyzed, was also estimated (Gregori et al., 2016; Seoane et al., 2022).

Comparative analyses between samples were performed using the QuasiComparer workflow (Seoane et al., 2022). Statistical analyses included ANOVA for comparative analysis between samples or sample variables (host species, dpi, rDl965 or Mut270Dl965), Shannon index and nucleotide diversity, calculated from reconstructed haplotypes, and principal component analysis (PCA) of all samples with all variables considered (Seoane et al., 2022).

## 3. Results

### 3.1. Viral genome quantification

Viral load in the brain of sea bass was tested in each experimental group by absolute RT-qPCR. Viral RNA1 and RNA2 were not detected in the negative control group, whereas in the groups infected with rDl965 or Mut270Dl965 they were quantified at the two sampling times considered (Figure 2; Supplementary material S2). RNA2 was more abundant than RNA1 in all samples, although the differences were not significant. The number of RNA1 and RNA2 copies of both viruses significantly increased from 1 to 5 dpi (*p* < 0.0001), reaching at the latter sampling time the maximum mean value of genome copies observed for both RNA1 (10.65 and 7.93 log copies/μg RNA for rDl965 and Mut270Dl965, respectively) and RNA2 (11.39 and 8.78 log copies/μg RNA, for rDl965 and Mut270Dl965, respectively). Moreover, the mean values obtained for RNA1 and RNA2 copy number in Mut270Dl965 were significantly lower than those obtained for the non-mutated virus at 5 dpi (*p* < 0.0001).

### 3.2. Genetic complexity of viral quasispecies in sea bass

Quasispecies characterization was firstly approached through analysis of individual samples (QuasiFlow analysis). After sequence filtering, between 6.87 × 10^5^ and 5.99 × 10^6^ paired reads per sample, and coverage between 2.69 × 10^4^ and 5.50 × 10^5^ nucleotides per nucleotide position, were obtained (Table 2; Supplementary material S3).

**Table 2:** Genetic complexity and heterogeneity values obtained for each of the RGNNV samples in each genomic segment.

RNA segment	Sample	Coverage	Mutation frequency^a^	H_n_^b^	H_SN_^c^	Nucleotide diversity	Recombination frequency^d^
RNA1	Dla_WT_1_r1	1.83 × 10^5^	4.30 × 10^−4^	3	0.04	8.59 × 10^−4^	7.51 × 10^−2^
	Dla_WT_1_r2	1.83 × 10^5^	4.43 × 10^−4^	8	0.23	8.86 × 10^−4^	3.46 × 10^−2^
	Dla_WT_1_r3	1.22 × 10^5^	-	-	0	-	5.32 × 10^−2^
	Dla_WT_5_r1	1.59 × 10^5^	1.99 × 10^−3^	16	0.1	3.95 × 10^−3^	1.63 × 10^−2^
	Dla_WT_5_r2	2.17 × 10^5^	7.79 × 10^−4^	12	0.12	1.53 × 10^−3^	2.03 × 10^−2^
	Dla_WT_5_r3	2.04 × 10^5^	5.37 × 10^−4^	6	0.32	1.07 × 10^−3^	2.06 × 10^−2^
	Dla_Mut_1_r1	5.11 × 10^4^	5.64 × 10^−4^	8	0.22	1.13 × 10^−3^	3.66 × 10^−2^
	Dla_Mut_1_r2	2.69 × 10^4^	2.97 × 10^−3^	9	0.31	5.46 × 10^−3^	1.61 × 10^−2^
	Dla_Mut_1_r3	3.63 × 10^4^	6.69 × 10^−4^	13	0.11	1.31 × 10^−3^	-
	Dla_Mut_5_r1	1.94 × 10^5^	5.86 × 10^−4^	11	0.37	1.14 × 10^−3^	9.75 × 10^−2^
	Dla_Mut_5_r2	1.91 × 10^5^	1.02 × 10^−3^	6	0.1	1.35 × 10^−3^	8.81 × 10^−2^
	Dla_Mut_5_r3	1.77 × 10^5^	4.30 × 10^−4^	6	0.43	8.59 × 10^−4^	1.42 × 10^−1^
	Sau_WT_1_r1	4.46 × 10^4^	6.44 × 10^−4^	18	0.04	1.28 × 10^−3^	1.83 × 10^−2^
	Sau_WT_1_r2	1.85 × 10^5^	1.43 × 10^−3^	9	0.06	2.86 × 10^−3^	3.23 × 10^−2^
	Sau_WT_1_r3	3.12 × 10^4^	7.16 × 10^−4^	9	0.1	1.41 × 10^−3^	2.80 × 10^−2^
	Sau_WT_5_r1	1.70 × 10^5^	1.66 × 10^−3^	7	0.36	3.28 × 10^−3^	4.41 × 10^−2^
	Sau_WT_5_r2	1.38 × 10^5^	1.20 × 10^−3^	7	0.42	2.39 × 10^−3^	4.98 × 10^−2^
	Sau_WT_5_r3	1.56 × 10^5^	7.25 × 10^−4^	12	0.08	1.45 × 10^−3^	5.68 × 10^−2^
RNA2	Dla_WT_1_r1	4.63 × 10^5^	1.92 × 10^−3^	4	0.01	3.84 × 10^−3^	5.15 × 10^−3^
	Dla_WT_1_r2	4.60 × 10^5^	6.46 × 10^−3^	4	0.08	1.29 × 10^−2^	1.52 × 10^−2^
	Dla_WT_1_r3	5.50 × 10^5^	7.22 × 10^−3^	3	0.02	1.44 × 10^−2^	8.74 × 10^−3^
	Dla_WT_5_r1	4.89 × 10^5^	-	-	-	-	9.03 × 10^−3^
	Dla_WT_5_r2	4.59 × 10^5^	-	-	-	-	2.08 × 10^−2^
	Dla_WT_5_r3	5.26 × 10^5^	-	-	-	-	1.14 × 10^−2^
	Dla_Mut_1_r1	3.86 × 10^5^	1.75 × 10^−3^	18	0.27	3.32 × 10^−3^	-
	Dla_Mut_1_r2	4.87 × 10^5^	1.35 × 10^−3^	14	0.03	2.68 × 10^−3^	1.06 × 10^−4^
	Dla_Mut_1_r3	2.79 × 10^5^	1.40 × 10^−3^	10	0.04	2.76 × 10^−3^	3.48 × 10^−4^
	Dla_Mut_5_r1	2.02 × 10^5^	1.93 × 10^−3^	13	0.04	3.74 × 10^−3^	1.40 × 10^−4^
	Dla_Mut_5_r2	5.30 × 10^5^	1.06 × 10^−2^	4	0.06	2.13 × 10^−2^	1.09 × 10^−4^
	Dla_Mut_5_r3	4.25 × 10^5^	-	-	-	-	5.84 × 10^−3^
	Sau_WT_1_r1	3.19 × 10^5^	1.62 × 10^−2^	4	0.06	1.80 × 10^−2^	2.58 × 10^−2^
	Sau_WT_1_r2	2.89 × 10^5^	3.21 × 10^−3^	5	0.03	6.28 × 10^−3^	8.92 × 10^−3^
	Sau_WT_1_r3	3.66 × 10^5^	1.55 × 10^−3^	9	0.11	3.06 × 10^−3^	2.41 × 10^−3^
	Sau_WT_5_r1	5.79 × 10^5^	1.14 × 10^−2^	3	0.03	2.23 × 10^−2^	2.21 × 10^−3^
	Sau_WT_5_r2	5.55 × 10^5^	-	-	-	-	1.70 × 10^−3^
	Sau_WT_5_r3	6.00 × 10^5^	9.54 × 10^−3^	3	0.02	1.91 × 10^−2^	2.36 × 10^−4^

^a^Mutation frequency (proportion of mutant nucleotides in a population) measured in mut/nt. ^b^Number of haplotypes. ^c^Normalized Shannon Index. ^d^Recombination frequency, expressed as number of recombinant reads divided by total number of reads (rec/rd).

We firstly analyzed the composition of mutations within RGNNV populations from sea bass, focusing on detecting possible point mutation biases. We observed a bias in base substitutions in the set of mutant spectra related to transition mutations. An overrepresentation of transitions was detected in all samples, in both segments, showing a Ts/Tv ratio between 3.68 and 16.21 (Table 2; Supplementary materials S3, S4), with high statistical significance (*p* < 0.01) (Supplementary material S5). The weight of transitions was not uniform in all samples. The abundance of transitions in the genome increased over time. However, there was a downward trend in the weight of transitions in Mut270Dl965 samples. Overall, Ts/Tv ratio was significantly lower in Mut270Dl965 than in rDl965 samples, especially at 1 dpi (Supplementary material S3). The lower Ts/Tv values were due to a considerable reduction in the number of T → C and C → T transitions (Supplementary materials S4, S5).

The complexity of RGNNV quasispecies was measured by estimates of mutation frequency and nucleotide diversity. Mutation frequency values ranged from 4.30 × 10^−4^ to 2.97 × 10^−3^ mut/nt in RNA1 and from 1.35 × 10^−3^ to 1.06 × 10^−2^ mut/nt in RNA2. Overall, mutation frequency values in Mut270Dl965 populations were significantly different from those in rDl965 populations, although with significant differences between viral segments. In virulent virus (rDl965) samples, there was a significant increase in mutation frequency in the RNA1 segment from 1 dpi (4.30–4.43 × 10^−4^ mut/nt) to 5 dpi (5.37 × 10^−4^–1.99 × 10^−3^ mut/nt). However, in low virulence virus (Mut270Dl965) samples, this parameter remained constant overtime, although a downward trend was detected at 5 dpi. Thus, rDl965 quasispecies were more complex at 5 dpi, whereas Mut270Dl965 complexity remained constant or even declined. In contrast, from 1 to 5 dpi, a decrease in genetic complexity was detected in RNA2 rDl965 quasispecies in sea bass. Mutation frequency ranged from 1.92 × 10^−3^ to 7.22 × 10^−3^ mut/nt at 1 dpi, dropping to values too low to be quantified by the workflow at 5 dpi (Table 2; Supplementary material S3). However, Mut270Dl965 RNA2 mutation frequency was the same at 1 dpi (1.35–1.75 × 10^−3^ mut/nt) as at 5 dpi (1.93 × 10^−3^-1.06 × 10^−2^ mut/nt) (Table 2; Supplementary material S3). Thus, the less virulent Mut270Dl965 virus quasispecies showed much greater genetic complexity in RNA2 than the virulent rDl965 virus quasispecies at 5 dpi.

The same patterns were observed when estimating genetic diversity. In the rDl965 samples, the RNA1 nucleotide diversity increased over time (8.59–8.86 × 10^−4^ and 1.07–3.95 × 10^−3^ at 1 and 5 dpi, respectively). However, RNA2 diversity decreased in samples collected at 5 dpi. Conversely, Mut270Dl965 nucleotide diversity remained constant for both segments at 1 and 5 dpi (Table 2; Supplementary material S3).

### 3.3. Recombination analysis in sea bass samples

Using the QuasiFlow tool, recombinant viruses were detected in the mutant spectra, and the recombination frequency was estimated (Seoane et al., 2022). The recombination frequency was different for each genomic segment. Thus, the estimated recombination frequency for RNA1 for all samples ranged from 10^−2^ to 10^−1^ recombinant reads per total number of reads (rec/rd), a wider range than that recorded for RNA2 (10^−4^–10^−2^ rec/rd). In general, the recombination events detected were homologous. Non-homologous recombination events were less abundant, being present mainly in rDl965-infected samples (Supplementary material S3).

Significant differences between the high virulent (rDl965) and low virulent quasispecies (Mut270Dl965) were detected in sea bass. Regarding RNA1, the recombination frequency in Mut270Dl965 populations was significantly higher (*p* < 0.01) than in rDl965 populations at 5 dpi (1.63–2.06 × 10^−2^ rec/rd for rDl965, and 8.81 × 10^−2^-1.42 × 10^−1^ for Mut270Dl965) (Table 2; Supplementary material S3). According to our results, a decrease in the generation of recombination events in RNA1 was observed from 1 to 5 dpi in the virulent quasispecies, whereas the number of recombinants increased considerably in the low virulent quasispecies in the same time interval. Regarding RNA2, virulent quasispecies underwent more recombination events than the less virulent quasispecies over time (*p* < 0.01). Recombination frequency in rDl965 samples was significantly higher (5.15 × 10^−3^–1.52 × 10^−2^ rec/rd at 1 dpi and 9.03 × 10^−3^–2.08 × 10^−2^ rec/rd at 5 dpi) than in Mut270Dl965 samples (1.06–3.48 × 10^−4^ rec/rd and 1.40 × 10^−4^–1.09 × 10^−2^ rec/rd at 1 and 5 dpi, respectively) (Table 2; Supplementary material S3).

**Figure 3:**
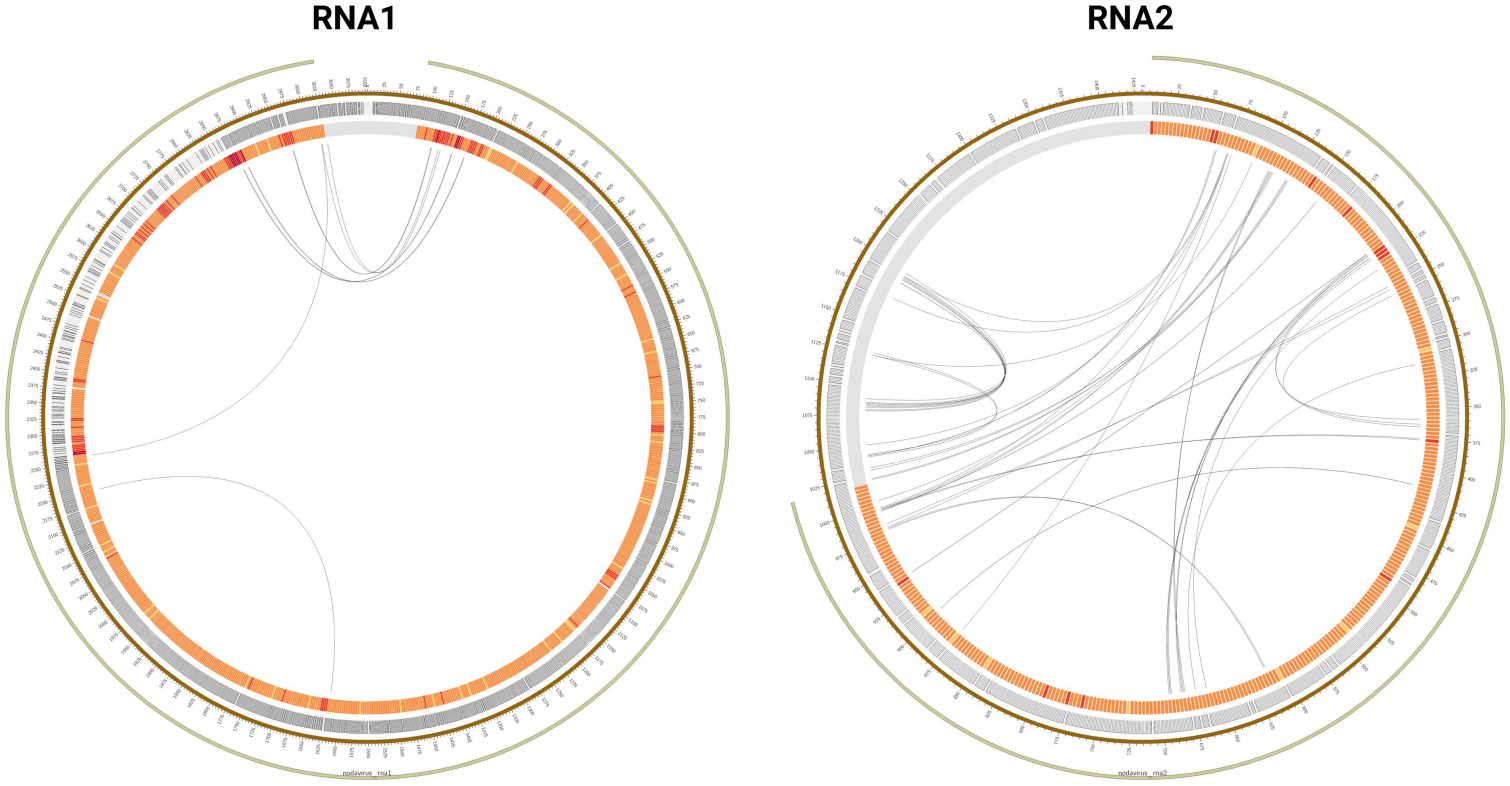
Mutation profile of the Dla_WT_1_r2 sample in the RNA1 and RNA2 segments. The mutation frequency of nucleotides and codons is expressed by a color code from yellow to red. Recombination points have been represented by black lines.

In addition, very characteristic recombination patterns between 3′ and 5′ ends of the RNA1 segment were observed (Figure 3; Supplementary material S6). Specifically, recombination originated in the region between nucleotide positions 2,850–3,050 and the region between positions 150–350. Nucleotide base coverage showed a characteristic pattern that could be correlated with recombination events, as it was significantly higher at 5′ and 3′ ends than in the rest of the genome. At some positions, coincident with recombination breakpoints, the coverage reached 10^6^ reads/base, i.e., ten-times higher than the average coverage estimated for the whole genome. Conversely, in the RNA2 segment, no recombination patterns common at all samples were detected.

### 3.4. Genetic heterogeneity of mutant spectra in sea bass

We evaluated the heterogeneity of the viral samples by reconstructing haplotypes and estimating the normalized Shannon index for each sample. Focusing on RNA1 segment, rDl965 quasispecies presented more haplotypes with a greater variety of mutations at 5 dpi (0–8 haplotypes per sample at 1 dpi and 6–16 haplotypes per sample at 5 dpi), constituting more complex and dynamic networks. However, the Shannon index did not vary significantly (0–0.23 for rDl965 at 1 dpi and 0.10–0.32 for rDl965 at 5 dpi) (Table 2; Supplementary material S3). This was because the dominant haplotypes exhibited similar abundance in the quasispecies in the 5 dpi samples (86–94%) as in the 1 dpi samples (90–99%) (Supplementary materials S7, S8). Mut270Dl965 quasispecies on the first day of infection were more heterogeneous (8–13 haplotypes per sample, and Shannon index of 0.11–0.31). In addition, non-dominant haplotypes of relative abundance (8–27%) were detected in these samples. However, the heterogeneity in the quasispecies of both viruses was similar at 5 dpi (Table 2; Supplementary materials S3, S7, S8).

**Figure 4:**
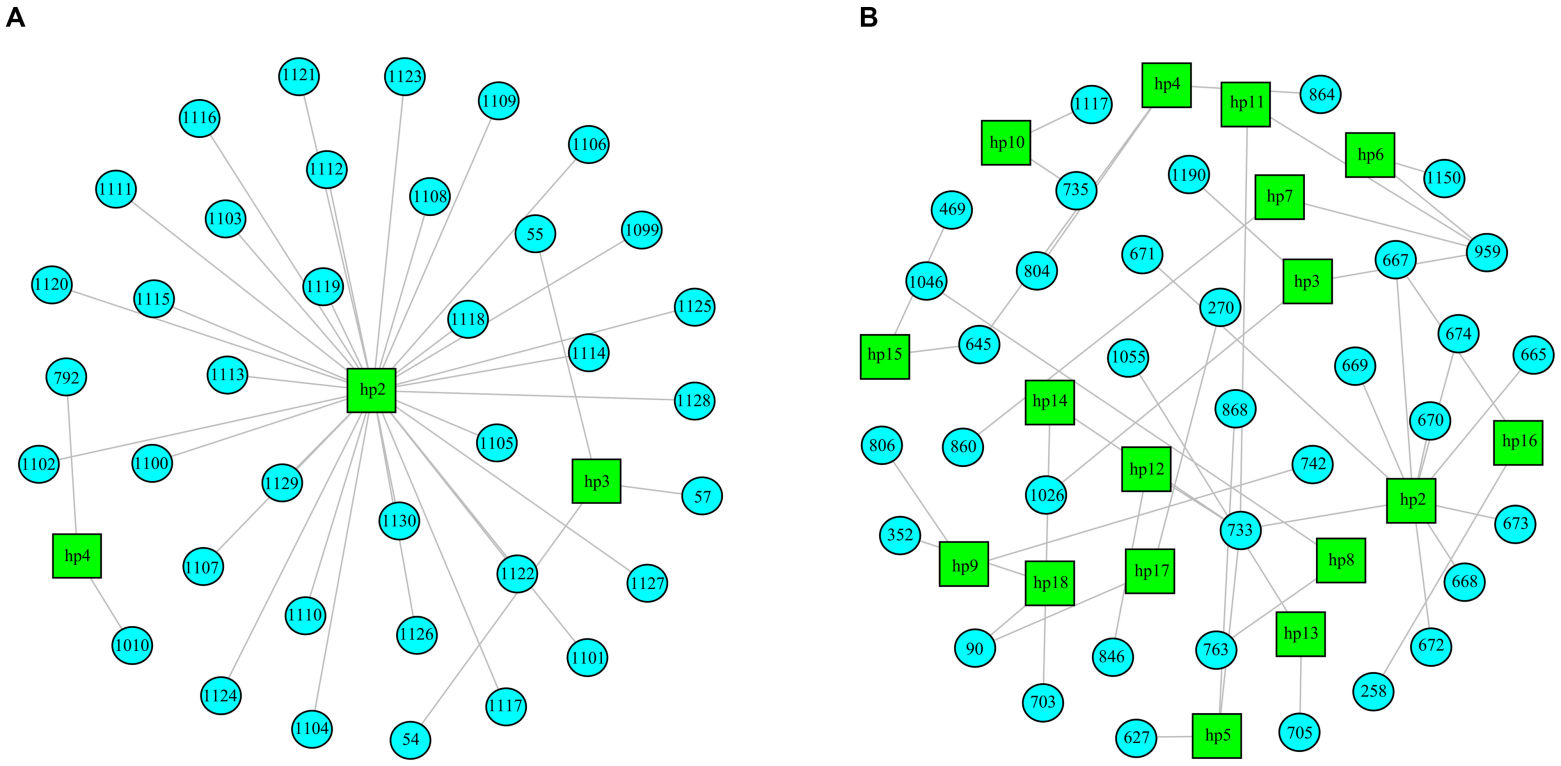
Distribution of haplotypes reconstructed from RNA2 segments of a rDl965 quasispecies [Dla_WT_1_r2 **(A)**] and a Mut270Dl965 quasispecies [Dla_WT_1_r1 **(B)**], both extracted from sea bass. Haplotypes are represented as green squares. Nucleotide changes for each haplotype are presented as blue circles with a numerical indicator referring to the position at which the change occurred with respect to the reference sequence.

Regarding the RNA2 segment, important differences in the heterogeneity of the samples according to their virulence and time of infection were observed. In rDl965 samples, genetic heterogeneity was very low (3–4 haplotypes per sample, and Shannon index of 0.01–0.08). At 5 dpi, QuasiFlow did not detect enough variability to reconstruct haplotypes for each quasispecies (Table 2; Supplementary material S3). As shown in Figure 4 and Supplementary material S8, Mut270Dl965 was more genetically heterogeneous in sea bass. The rDl965 quasispecies at 1 dpi were characterized by a few haplotypes, of which the dominant haplotype constituted 99% of the sequences. By contrast, Mut270Dl965 quasispecies contained a greater number of haplotypes (10–18). However, it is noteworthy that the haplotypes in the Mut270Dl965 samples were characterized by few mutations and very low relative abundance, except for sample Dl_Mut_r1, where one haplotype with a frequency above 20% was recorded (Supplementary material S7). On the other hand, a decrease in heterogeneity in samples collected at 5 dpi (0–13 haplotypes per sample and 0–0.06 Shannon index) was also observed. Thus, a decrease in haplotype diversity was observed with the course of infection for both viruses, although less pronounced for the low virulent mutant (Table 2; Supplementary material S3).

In addition, rare haplotypes containing a considerable number of mutations were detected in several quasispecies regardless of its virulence, suggesting that they may have appeared as a result of a hypermutation phenomenon (Supplementary material S7).

### 3.5. Characterization of rDl965 quasispecies in sea bream

To test the hypothesis that NNV virulence may be associated with genetic complexity and heterogeneity, we analyzed rDl965 quasispecies in sea bream. In this way, we could evaluate differences in the mutant spectra of the same virus in two hosts having different degrees of susceptibility. The analyses in sea bream were performed following the same procedures described for sea bass samples.

**Figure 5:**
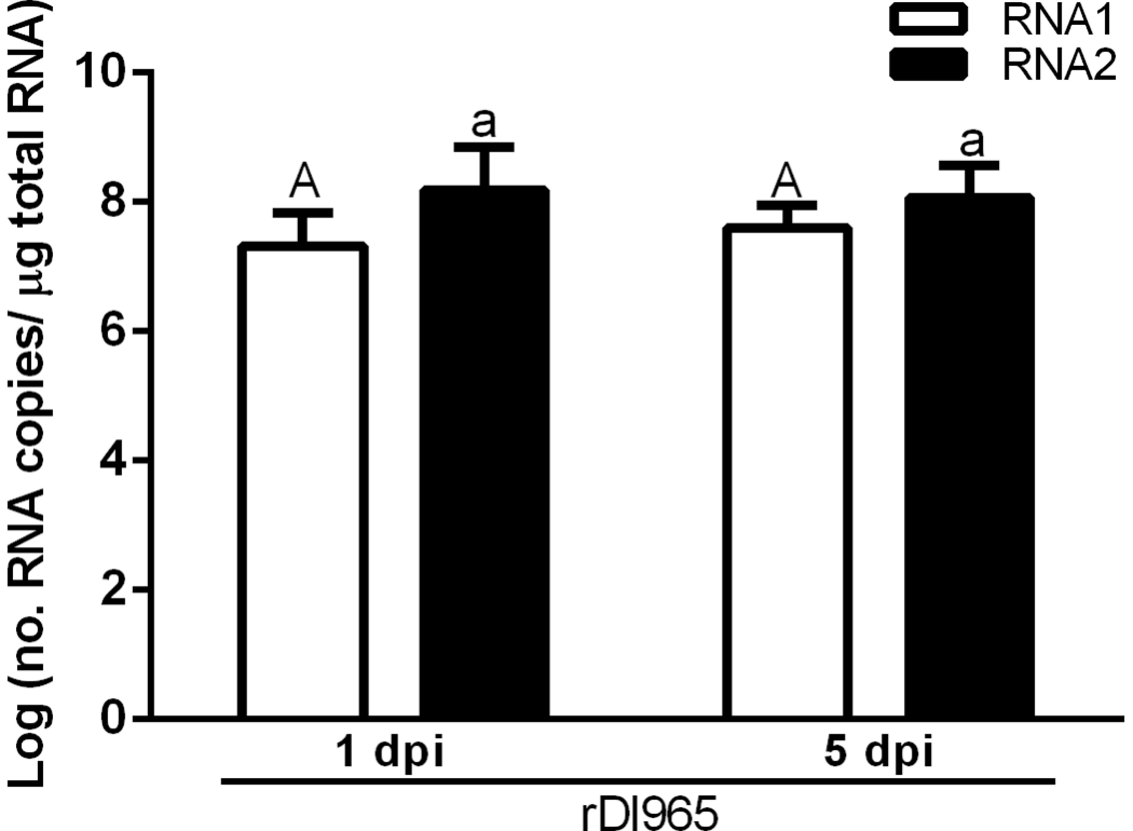
Average number of RNA1 and RNA2 molecules per μg of total RNA (mean ± standard deviation) in brain of sea bream specimens infected with rDl965. Different uppercase and lowercase letters indicate significant differences in RNA1 or RNA2 copy numbers, respectively, between experimental groups.

First, viral load of NNV in sea bream brain was determined by RT-qPCR. rDl965 RNA1 and RNA2 copy number in sea bream was similar at the two times sampled, being within the same ranges as sea bass rDl965 samples at 1 dpi (8.25 and 8.91 log copies/μg RNA for RNA1 and RNA2, respectively) (Figure 5; Supplementary material S2).

The composition of mutations in the rDl965-sea bream samples was similar to quasispecies detected in sea bass. A significant (*p* < 0.01) overrepresentation of transitions that maintained a high Ts/Tv ratio (6.70–13.94) was recorded (Table 2; Supplementary materials S3–S5). The Ts/Tv ratio was lower than that estimated in rDl965-bass samples, but not to a significant extent.

Overall, in the mutant spectra in sea bream, the mutation frequency was significantly (*p* < 0.01) higher than that in sea bass (Table 2; Supplementary material S3). Values remain constant for both RNA1 (6.44 × 10^−4^-1.43 × 10^−3^ and 7.25 × 10^−4^-1.66 × 10^−3^ mut/nt at 1 and 5 dpi, respectively) and RNA2 (1.55 × 10^−3^-1.62 × 10^−2^ and 9.54 × 10^−3^-1.14 × 10^−2^ mut/nt at 1 and 5 dpi, respectively), indicating that complexity and diversity of rDl965 quasispecies in sea bream are reached earlier, during the first day of infection. On the other hand, the frequency of recombination events estimated for RNA1 was similar to that calculated for the virus in sea bass. However, in the RNA2 from samples collected at 5 dpi, the number of recombination events was lower than in samples from sea bass at the same time (Table 2; Supplementary material S3). In addition, the same recombination patterns between the 5′ and 3′ ends of the RNA1 genomic segment were also observed (Supplementary material S6).

The analysis of the reconstructed haplotypes and the interaction networks for each quasispecies showed a different distribution in each segment. In RNA1, more rDl965 haplotypes were observed in sea bream at 1 dpi (9–18 haplotypes per sample) than at 5 dpi (7–12 haplotypes per sample). However, the Shannon index increased from 1 dpi (0.04–0.10) to 5 dpi (0.08–0.42) (Table 2; Supplementary material S3), due to the distribution and abundance of the different haplotypes. Samples at 1 dpi had more haplotypes, but the dominant haplotype accounted for 98–99% of the isolated sequences. In contrast, samples at 5 dpi had slightly fewer haplotypes, but dominant haplotypes were less abundant (69–96%) and coexisted with relatively abundant haplotypes (3–30%) (Supplementary material S7). There were no significant differences with rDl965 samples in sea bass. However, rDl965 RNA2 quasispecies in sea bream were more heterogeneous than those in sea bass, containing a greater number and abundance of haplotypes, especially at 5 dpi (Table 2; Supplementary materials S3, S7, S8).

**Figure 6:**
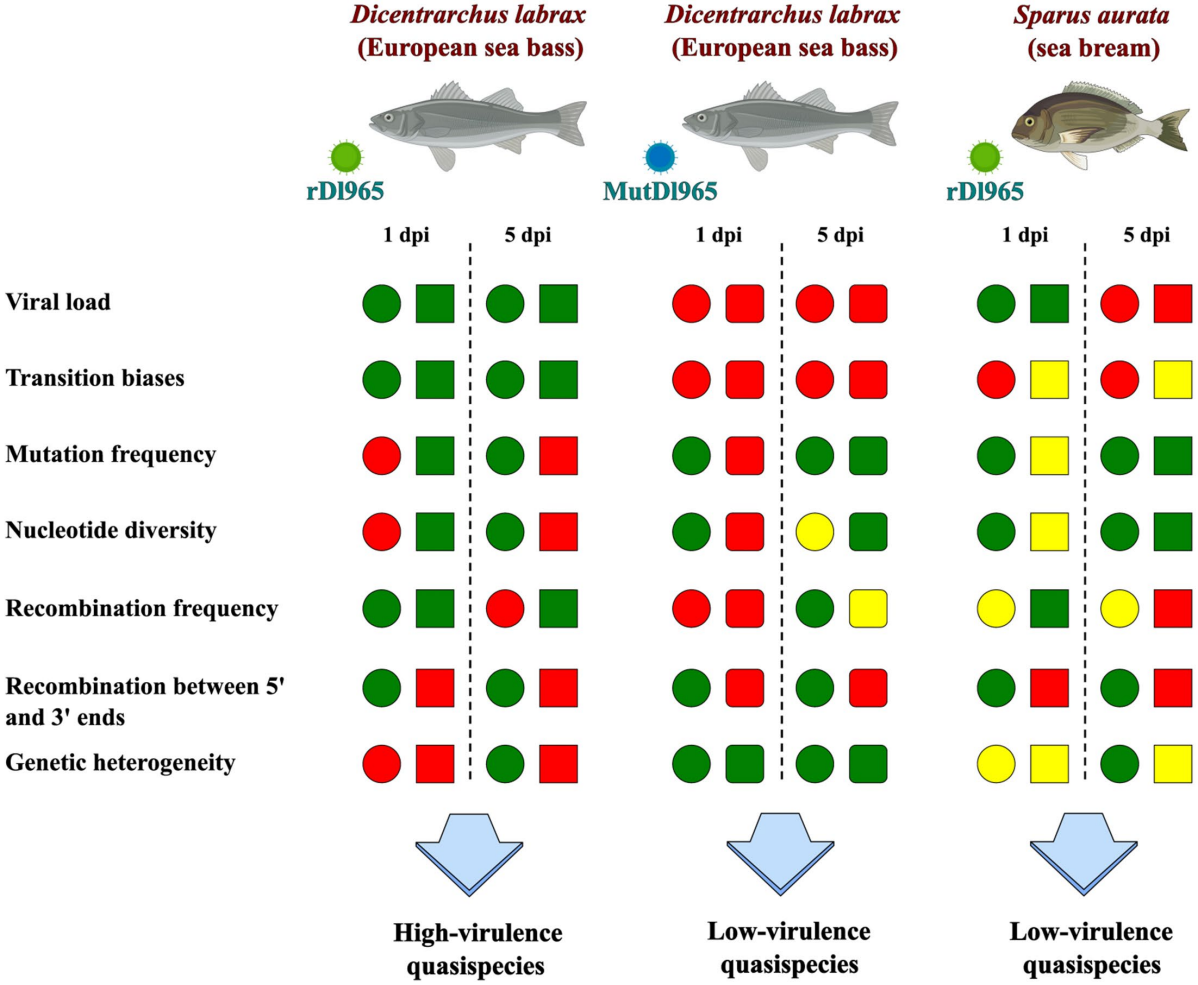
Summary of the differences observed between low and high virulence RGNNV quasispecies. Comparative results are shown for each genomic segment between the quasispecies at 5 and 1 dpi. Color coding is shown with respect to the rDl965 virus quasispecies in sea bass, with green being a higher value, red a lower value, and yellow a similar value. For each estimate analyzed, the RNA1 segment is shown as a circle, the RNA2 segment whose sequence is that of the rDl965 virus is shown as a square, and the RNA2 carrying the G829A mutation is shown as a square with rounded corners.

The genetic characteristics of the quasispecies recorded in sea bream and sea bass are summarized in Figure 6.

### 3.6. Multivariate analysis of virulent and non-virulent RGNNV infections in sea bass and sea bream

**Figure 7:**
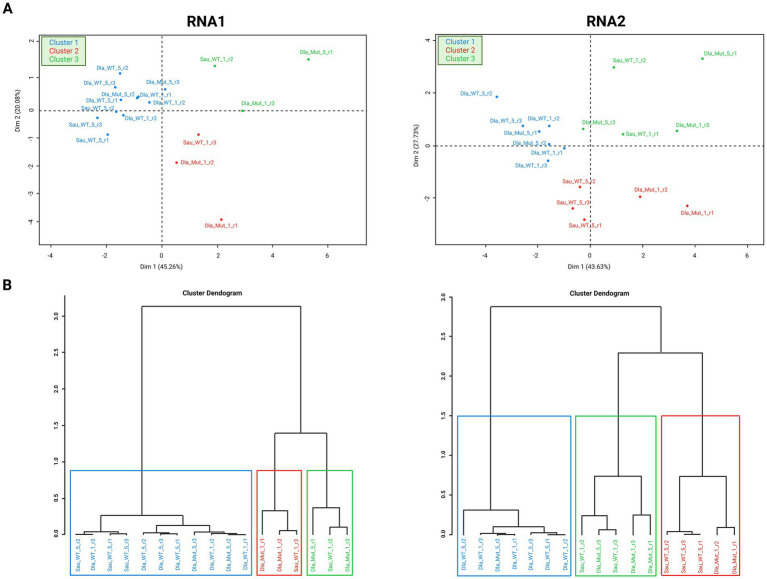
Principal component analysis (PCA). Analyses were performed separately for RNA1 (left) and RNA2 (right) segments. **(A)** distribution and clustering of each sample analyzed by PCA in dot plots. **(B)** Distribution and clustering of the samples analyzed by PCA in dendrograms.

Comparative statistical analysis was carried out with the QuasiComparer workflow. We tested significant variations in the structure and variability of the NNV quasispecies analyzed. A principal component analysis (PCA) considering all samples analyzed was performed for RNA1 and RNA2 segments together and separately (Figure 7). A total of 11 variables were included, such as Ts/Tv ratio, Shannon index, transitions, transversions, ORF mutation frequency, SNPs or recombination events (Supplementary material S9). The genetic variables used to describe each sample in the PCA were defined in each dimension of the two-dimensional space.

We first performed the PCA by combining the two genomic segments. Almost all of the RNA1 sequences in the samples were grouped into two clusters, separate from the RNA2 sequences. Only two RNA1 samples were grouped with the RNA2 samples in the cluster. Samples isolated from different hosts were mixed. The RNA2 sequences were grouped into a single cluster. Mutation and recombination frequencies, as well as nucleotide diversity, were the most defining variables of this cluster. Thus, these variables were in RNA2 considerably different than in RNA1 in the RGNNV quasispecies (Supplementary material S9).

We then performed two PCAs, one for each segment. PCA representations for RNA1 and RNA2 segments did not show well-defined clustering according to the features selected to classify each sample (host, dpi and virus). Samples grouped into three clusters (Figure 7). Cluster 1comprised all rDl965 samples in sea bass, and was recorded for both viral segments. In both segments, no significant differences were observed between virulent quasispecies of RGNNV in sea bass at 1 and 5 dpi. All Mut270Dl965 sea bass samples and rDl965 in sea bream samples were grouped in two clusters (2 and 3) and separated from rDl965 quasispecies in sea bass (Cluster 1). Only two Mut270Dl965 quasispecies were also included in RNA2 Cluster 1, and five were included in RNA1 Cluster 1 (two samples of Mut270Dl965 from sea bass and three samples of rDl965 from sea bream). These results suggest that Mut270Dl965 quasispecies in sea bass are more similar to rDl965 quasispecies in sea bream (low virulent) than to rDl965 quasispecies infecting sea bass (highly virulent). PCA analysis showed that the RNA2 of all rDl965 samples in sea bream at 1 dpi are significantly different from those detected at 5 dpi. These results contrast with those observed for RNA1 quasispecies.

Examining the distribution of the variables along the PCA axes, we found that the most important variable characterizing cluster 1 was the Ts/Tv ratio. In addition, the number of recombination events and the recombination frequency were also relevant in the PCA analysis of the RNA2 segment (Supplementary material S9).

## 4. Discussion

The role of amino acid at position 270 within the RGNNV capsid protein as a virulence determinant to sea bass has been recently shown. A non-synonymous mutation at this position (G829A), which replaces serine by asparagine (Ser270Asp), causes a decrease in virulence to sea bass (Moreno et al., 2019). In RNA viruses, one or a few substitutions can alter adaptation and pathogenesis (Domingo et al., 2006). In the present study, we analyze how this mutation affects the structure and evolution of viral quasispecies *in vivo*.

The number of RNA1 and RNA2 copies in brain of sea bass and sea bream was quantified by absolute RT-qPCR at 1 and 5 dpi. The number of viral genome copies is higher in RGNNV-infected sea bass (highly virulent phenotype) than in Mut270Dl965-sea bass (low virulent phenotype). These results suggest a positive correlation between viral load and virulence of NNV. However, the virulence of a virus is not necessary associated with replicating capacity and survival ability, i.e., its viral fitness (Domingo et al., 2008). The correlation between virulence and fitness may depend on complex virus-host interactions, as well as between coexisting variants in the host (Furió et al., 2012). At 1 dpi, the viral load in sea bream did not vary significantly compared to RNA levels detected in sea bass. However, at 5 dpi, it was observed that the viral load in sea bream was much lower than in sea bass. RGNNV infection in sea bream is asymptomatic, or generates mild symptoms, with no or very low mortality rate in juveniles (Castric et al., 2001; Valero et al., 2015), due to an efficient immune response (Poisa-Beiro et al., 2008; Pereiro et al., 2023).

In this work we analyze the genetics of RGNNV focusing on each genomic segment separately. Although there was a downward trend in the RNA1 segment, there was no statistically significant difference in the estimated viral load in the RNA1 and RNA2 segments. In NNV, there is a modulation in the replication of genomic segments that determines the load of each segment throughout the infectious cycle (Eckerle and Ball, 2002; Lindenbach et al., 2002; Souto et al., 2018). However, despite the similarities in the viral load, they had important differences in genetic variability between two segments. Focusing on rDl965 sea bass samples, the results suggest that the variation and diversification of each genomic segment is different in quasispecies throughout the infection. This finding was corroborated by PCA analyses performed with both segments. In segmented viruses, each segment undergoes a different genetic selection and diversification, allowing studying the evolutionary history of each segment independently (Rambaut et al., 2008; Freire et al., 2015). In our samples, RNA1 diversification was continuous along the infection in sea bass during the first 5 days. In contrast, RNA2 tended toward homogeneity over time. These current findings contrast with evolutionary analyses reporting that RGNNV genome undergoes strong purifying selection pressure. However, they did show that the rate of evolution in the RNA1 was significantly higher than in the RNA2 segment (Panzarin et al., 2012).

Sequence analyses revealed that both genomic segments showed high genetic variability. Low-frequency mutations can be detected thanks to the in-depth assessment of the population structure and complexity of viral quasispecies enabled by NGS bioinformatics analysis tools (Pérez-Losada et al., 2020). However, NGS produces millions of error-prone short reads, generating artifacts from sequencing errors that may not be distinguishable from real mutations, especially low-frequency ones (Gregori et al., 2014, 2016; Knyazev et al., 2020). QuasiFlow performs a stringent filtering process on raw NGS reads that removes reads with sequence changes caused by artificial mutations, providing reliable detection of low-frequency genetic variations and ensuring the reliability of downstream analyses (Seoane et al., 2022).

In this report we suggest that changes in the genetic structure of *in vivo* quasispecies of a nodavirus may underlie differences in host virulence. Previously, different types of RGNNV quasispecies have been shown to influence immunopathogenesis in a host undergoing persistent infection. These quasispecies possessed different characteristics, including localization within the host, infectivity or suppression of host immunity (Chen et al., 2020). Changes in genetic heterogeneity can dramatically affect virulence in a viral population (Vignuzzi et al., 2006). In this regard, we show that a single point mutation in the consensus sequence of the RNA2 segment, which encodes the capsid protein, caused a significant change in the quasispecies, affecting the virulence of the virus.

Focusing on the RNA2 segment, low virulent Mut270Dl965 virus generated a more complex and genetically heterogeneous quasispecies. These data are surprising, given that a genetically homogeneous quasispecies may be more vulnerable and fail to overcome selection pressure. The generation of highly variable quasispecies allows viruses to adapt to different environments (Domingo et al., 2012; Domingo and Perales, 2019; Donohue et al., 2019; Delgado et al., 2021). A larger reservoir of mutations and haplotypes in a quasispecies favors not only host adaptation but can generate new phenotypes that positively affect virulence (Moreno et al., 2017). Previously, a mutant poliovirus with higher copy fidelity was reported to generate a more homogeneous quasispecies than the wild-type virus, resulting in the loss of its ability to infect the central nervous system in mice (Pfeiffer and Kirkegaard, 2005; Vignuzzi et al., 2006). In Chikungunya virus (CHIKV), induction of high-fidelity mutations produced more diverse and virulent virus populations, whereas low-fidelity mutations generated less diverse populations that produce attenuated disease (Riemersma et al., 2019).

Despite higher haplotype diversity in the low-virulence virus populations, a tendency towards homogeneity was still detected, as revealed by a normalized Shannon index below 0.5. More haplotypes emerged in the low virulent quasispecies, although the frequency of the dominant haplotype did not change significantly compared to the virulent populations. Only in the Dla_Mut_1_r1 sample a considerable increase in heterogeneity was detected, as a second haplotype emerged with a relatively high frequency (27%). As it has been described for the virulent virus samples, although with less intensity, this trend toward homogeneity increased at 5 dpi. The evolutionary history of a quasispecies is determined not by the characteristics of the fittest variant or master sequence, but by the set of variants that make up the mutant spectrum (Domingo et al., 2006; Domingo, 2016). Selection favors those genotypes with a faster replication rate, which also implies the production of new variants with lower fitness (Wilke et al., 2001). It should be noted that the samples collected at 1 and 5 dpi belong to different individuals. Therefore, to understand the evolution of RGNNV quasispecies in a host, it would be necessary to investigate it in the same specimen during the course of the infection.

Virus-host interactions must be considered when determining the fate of a viral population. Changes in genetic heterogeneity are not only generated by disparity in the degree of genetic diversity, but can also be caused by differences in genomic patterns. Serial passaging assays in cell culture with an attenuated strain of mumps virus showed that the significant decrease in its neurovirulence came from changes in the proportion of mutations. Depending on the genome site, increases and decreases in the genetic heterogeneity of the attenuated quasispecies were found (Sauder et al., 2006). It has been reported that the pathogenesis of Newcastle disease virus (NDV) can be modulated by the proportion of avirulent and virulent genomes coexisting within the quasispecies and their interactions (Meng et al., 2016). These changes may result in purifying selection in the coding regions of virulent genotypes, affecting genetic diversity in this region (Jadhav et al., 2020).

Changes in virulence were associated with variations in the entire quasispecies, not only in the RNA2 segment. In RNA1, significant variations in recombination and mutation frequencies were detected in Mut270Dl965 samples with respect to rDl965 samples. Furthermore, in the samples at 1 dpi we found that in the Mut270Dl965 populations the nucleotide and haplotype diversity was higher. Therefore, our results suggest that the G829A mutation in RNA2 may affect the evolution of the viral quasispecies in the RNA1 segment. In segmented viruses, although each segment exhibits specific variability, the interaction between them influences their evolution (Pontremoli et al., 2018).

The properties of rDl965 quasispecies were evaluated in sea bream, a host less susceptible to RGNNV. PCA analysis revealed that rDl965 populations isolated from sea bream were more closely related to the low virulent Mut270Dl965-sea bass quasispecies than to the high virulent rDl965-sea bass quasispecies, especially with respect to the RNA2 segment. These results suggest that the genetic variability and evolution of a mutant spectrum may be primarily associated with its virulence. Similarities existed between quasispecies inhabiting environments exposed to different selection pressures. In Mut270Dl965 quasispecies, the Ser270Asp mutation may alter the interaction of the capsid protein with the cellular receptor (Moreno et al., 2019), which could constitute a bottleneck leading to quasispecies restriction, since it would impair RGNNV infection. Bottlenecks can abruptly change the structure and variability of a viral population (Pfeiffer and Kirkegaard, 2005; Cortey et al., 2018; Kadoya et al., 2020), establishing a founder effect that can lead to increased genetic diversity within quasispecies. This hypothesis could explain the increase in genetic variability and diversity in mutant virus populations at 1 dpi. The transcription of genes related to the innate immune response induced by Mut270Dl965 is lower than that triggered by the wild-type virus (Moreno et al., 2019). This may result in weaker immune-mediated selection pressure and lead to greater diversification of viral populations. However, in sea bream, RGNNV infection generates a strong immune response (Pereiro et al., 2023). Sea bream is considered a natural reservoir of NNV (Castric et al., 2001; Chaves-Pozo et al., 2012; Valero et al., 2015). Nodavirus quasispecies may show different characteristics in healthy hosts than in hosts undergoing severe acute infection (Chen et al., 2020). This means that RGNNV populations present in potentially resistant hosts could develop their mutant clouds under strong immune-mediated pressure, acquiring different characteristics than if they were in a host susceptible to acute infection. Similarly, it would be desirable to analyze a highly virulent isolate for sea bream, in order to compare the quasispecies of virulent isolates of NNV between the two hosts.

Recombination events were very frequent in both segments in all samples analyzed. The frequency of recombination in the RNA1 sequence was considerably higher than the mutation frequency. Our results suggest the importance of recombination in the origin of genetic variability and the constitution of quasispecies throughout host infection. This is one of the first studies addressing the analysis of genetic material exchange events in NNV at an intrahost level. In NNV, genetic exchange and reassortment between viral species have been frequently described (Toffolo et al., 2007; Panzarin et al., 2012, 2014; Souto et al., 2015a; Toffan et al., 2017; Ariff et al., 2019; Volpe et al., 2020). However, the effects that recombinant haplotypes within an RGNNV quasispecies may have on pathogenesis are still unknown.

Cumulative recombination events were observed between the 5′ and 3′ ends of the RNA1 segment in all samples analyzed. These patterns of recombination events in the RNA1 segment could suggest the synthesis of circular RNAs (circRNAs) in RGNNV. CircRNAs can be generated through backsplicing (Choudhary et al., 2021; Tan et al., 2021), a process that has not yet been described in NNV. The generation of circRNAs is present in both, DNA and RNA viruses (Zhang et al., 2022). In herpesviruses (double-stranded DNA viruses), the generation of circRNAs has been observed in response to activation of the lytic cycle of infection. These molecules may participate in the regulation of processes such as cell proliferation or host innate immune response (Tagawa et al., 2018; Tan et al., 2021). They have only been described in some negative-stranded RNA viruses encoding viral circRNAs. The synthesis of circRNA molecules has been described in sarbecoviruses (Cai et al., 2021), and it is suggested that they may be involved in the generation of homologous recombinants (Hassanin et al., 2022). CircRNAs has been discovered in a fish dsRNA virus, grass carp reovirus (GCRV). Some of these molecules were formed by alternative splicing, and intervened by suppressing GCRV replication (Pan et al., 2021). However, we cannot confirm, beyond the results obtained by bioinformatics analysis, the generation of circRNAs in RGNNV. The role that this process may play in viral pathogenesis is also undetermined. Further work on the detection and functional characterization of circRNAs is needed in future studies.

## Data availability statement

The data presented in the study are deposited in the SRA repository, accession number PRJNA942601, and can be found at: https://www.ncbi.nlm.nih.gov/sra/PRJNA942601.

## Ethics statement

The animal study was reviewed and approved by Bioethics Committee of Junta de Andalucia (number: 09/08/2019/136).

## Author contributions

AG-P, JB, EG-R, MA, and LD-M: conceptualization and design of the study. LD-M, PM, and SO-D-C: investigation and formal analysis. LD-M and SO-D-C: bioinformatics and statistical analysis. SO-D-C and AG-P: writing. All authors contributed to manuscript revisions, read, edition, and approved the submitted version.

## Funding

This study has been supported by the project PID2020-115954RB-100/AEI/10.13039/501100011033 (Spanish Government). JB and AG-P are grateful to the Plan Propio of the University of Malaga.

## Conflict of interest

The authors declare that the research was conducted in the absence of any commercial or financial relationships that could be construed as a potential conflict of interest.

## Publisher’s note

All claims expressed in this article are solely those of the authors and do not necessarily represent those of their affiliated organizations, or those of the publisher, the editors and the reviewers. Any product that may be evaluated in this article, or claim that may be made by its manufacturer, is not guaranteed or endorsed by the publisher.

## Supplementary material

The Supplementary material for this article can be found online at: https://www.frontiersin.org/articles/10.3389/fmicb.2023.1182695/full#supplementary-material
