# Supplementary material for: The genetic variability and evolution of red-spotted grouper nervous necrosis virus quasispecies can be associated with its virulence
Source: Front Microbiol. 2023 Jun 15;14:1182695. doi: 10.3389/fmicb.2023.1182695 (PMC10308047; doi:10.3389/fmicb.2023.1182695)
Supplement: Supplementary file 1 [file Data_Sheet_1.zip › Supplementary Material S1.docx]

Supplementary Material S1

**The genetic variability and evolution of red-spotted grouper nervous necrosis virus quasispecies can be associated with its virulence**

**Sergio Ortega-del Campo, Luis Díaz-Martínez, Patricia Moreno, Esther García-Rosado, M. Carmen Alonso, Julia Béjar* and Ana Grande-Pérez***

*** Correspondence:** Corresponding Author: bejar@uma.es & agrande@uma.es

**Supplementary Table 1.** Primers used in this study.

| Name | Sequence 5'-3' | Product length (bp) | Usage | Reference^a^ |
| --- | --- | --- | --- | --- |
| T7_5’RNA1_965 | GGATCCCCGCGGTAATACGACTCACTATAGGTAACATCCCTTTCTTGCTCTGTT | 3,104 | RNA1 sequencing | Moreno et al., (2019) |
| 3’RNA1_965 | GGCGCCGAAGCGTAGGACAGCATAAAGC |  |  |  |
| T7_5’RNA2_965 | GGATCCCCGCGGTAATACGACTCACTATAGGTAATCCATCACCGCTTTGCAATC | 1,432 | RNA2 sequencing | Moreno et al., (2019) |
| 3’RNA2_965 | GGCGCCGAGTTGAAAAGCGATCAGCGG |  |  |  |
| Mut270Dl965 | TCCGCTGTCTATTGACTACAACCTTGGAACTGGAG |  |  |  |
| RG-RNA1-F | GGCTCAGATCTGGTAATGTTTCAA | 63 | RGNNV qPCR | Lopez-Jimena et al., (2011) |
| RG-RNA1-R | CAAAGCCAAGGGAAGAAGCA |  |  |  |
| RG_965_RNA2 F4 | ACCGTCCGCTGTCTATTGACTA | 122 |  | Moreno et al., (2016) |
| 35 RG_965_RNA2 R1 | CAGATGCCCCAGCGAAACC |  |  |  |

^a^References of the studies that designed each of the primers used in our work.
