# Supplementary material for: The genetic variability and evolution of red-spotted grouper nervous necrosis virus quasispecies can be associated with its virulence
Source: Front Microbiol. 2023 Jun 15;14:1182695. doi: 10.3389/fmicb.2023.1182695 (PMC10308047; doi:10.3389/fmicb.2023.1182695)
Supplement: Supplementary file 1 [file Data_Sheet_1.zip › Supplementary Material S2.docx]

Supplementary Material S2

**The genetic variability and evolution of red-spotted grouper nervous necrosis virus quasispecies can be associated with its virulence**

**Sergio Ortega-del Campo, Luis Díaz-Martínez, Patricia Moreno, Esther García-Rosado, M. Carmen Alonso, Julia Béjar* and Ana Grande-Pérez***

*** Correspondence:** Corresponding Author: bejar@uma.es & agrande@uma.es

**Supplementary Table 1.** Viral load (RNA copy number) quantified in red-spotted grouper nervous necrosis virus (RGNNV) samples in sea bass.

| **Samples^a^** | **Number of viral RNA copies** | | | |
| --- | --- | --- | --- | --- |
|  | **RNA1 copies^b^** | | **RNA2 copies** | |
|  | **Per sample** | **Average** | **Per sample** | **Average** |
| **Dla_WT_1_r1** | 3.66 × 10^8^ | 1.70 × 10^8^ | 1.78 × 10^9^ | 8.22 × 10^8^ |
| **Dla_WT_1_r2** | 1.23 × 10^8^ |  | 5.53 × 10^8^ |  |
| **Dla_WT_1_r3** | 2.02 × 10^7^ |  | 1.32 × 10^8^ |  |
| **Dla_WT_5_r1** | 3.19 × 10^10^ | 4.71 × 10^10^ | 1.77 × 10^11^ | 2.55 × 10^11^ |
| **Dla_WT_5_r2** | 5.84 × 10^10^ |  | 3.40 × 10^11^ |  |
| **Dla_WT_5_r3** | 5.10 × 10^10^ |  | 2.48 × 10^11^ |  |
| **Dla_Mut_1_r1** | 4.25 × 10^5^ | 5.90 × 10^5^ | 3.87 × 10^6^ | 3.99 × 10^6^ |
| **Dla_Mut_1_r2** | 1.06 × 10^6^ |  | 3.92 × 10^6^ |  |
| **Dla_Mut_1_r3** | 2.89 × 10^5^ |  | 4.20 × 10^6^ |  |
| **Dla_Mut_5_r1** | 6.88 × 10^7^ | 8.80 × 10^7^ | 5.51 × 10^8^ | 6.40 × 10^8^ |
| **Dla_Mut_5_r2** | 7.87 × 10^7^ |  | 4.31 × 10^8^ |  |
| **Dla_Mut_5_r3** | 1.16 × 10^8^ |  | 9.38 × 10^8^ |  |

^a^The average viral RNA copy number was calculated as a function of sampling day, as well as virus and host type.

^b^The number of copies of each segment in each viral RNA sample was estimated.

**Supplementary Table 2.** Viral load (RNA copy number) quantified in red-spotted grouper nervous necrosis virus (RGNNV) samples in sea bream.

| **Samples^a^** | **Number of viral RNA copies** | | | |
| --- | --- | --- | --- | --- |
|  | **RNA1 copies^b^** | | **RNA2 copies** | |
|  | **Per sample** | **Average** | **Per sample** | **Average** |
| **Sau_WT_1_r1** | 6.79 × 10^7^ | 3.71 × 10^7^ | 4.81 × 10^7^ | 4.85 × 10^8^ |
| **Sau_WT_1_r2** | 6.24 × 10^6^ |  | 1.36 × 10^9^ |  |
| **Sau_WT_1_r3** | **-** |  | 4.87 × 10^7^ |  |
| **Sau_WT_5_r1** | 8.74 × 10^7^ | 5.23 × 10^7^ | 3.61 × 10^8^ | 1.98 × 10^8^ |
| **Sau_WT_5_r2** | 1.72 × 10^7^ |  | 3.58 × 10^7^ |  |
| **Sau_WT_5_r3** | - |  | - |  |

^a^The average viral RNA copy number was calculated as a function of sampling day, as well as virus and host type.

^b^The number of copies of each segment in each viral RNA sample was estimated.
