# Supplementary material for: The genetic variability and evolution of red-spotted grouper nervous necrosis virus quasispecies can be associated with its virulence
Source: Front Microbiol. 2023 Jun 15;14:1182695. doi: 10.3389/fmicb.2023.1182695 (PMC10308047; doi:10.3389/fmicb.2023.1182695)
Supplement: Supplementary file 1 [file Data_Sheet_1.zip › Supplementary Material S4.docx]

Supplementary Material S4

**The genetic variability and evolution of red-spotted grouper nervous necrosis virus quasispecies can be associated with its virulence**

**Sergio Ortega-del Campo, Luis Díaz-Martínez, Patricia Moreno, Esther García-Rosado, M. Carmen Alonso, Julia Béjar* and Ana Grande-Pérez***

*** Correspondence:** Corresponding Author: bejar@uma.es & agrande@uma.es

**Supplementary Table 1.** Summary of mutations present in RNA1 quasispecies of red-spotted grouper nervous necrosis virus RGNNV in sea bass.

| **Sample** | | **SNP** | | | | | | | | | | | | | | **InDels** | | | | |
| --- | --- | --- | --- | --- | --- | --- | --- | --- | --- | --- | --- | --- | --- | --- | --- | --- | --- | --- | --- | --- |
|  |  | **Transitions (Ts)** | | | | **Transversions (Tv)** | | | | | | | | | **Total** | **Insertions** | **Deletions** | | | **Total** |
|  |  | A → G | C → T | G → A | T → C | A → C | A → T | C → A | C → G | G → C | G → T | T → A | T → G |  | |  | |  |  | |
|  | Dla_WT_1_r1 | 401 | 732 | 619 | 699 | 20 | 100 | 28 | 4 | 3 | 22 | 14 | 5 | 2647 | | 5 | | 21 | 26 | |
|  | Dla_WT_1_r2 | 351 | 646 | 547 | 614 | 19 | 133 | 38 | 2 | 10 | 34 | 19 | 17 | 2430 | | 11 | | 28 | 39 | |
|  | Dla_WT_1_r3 | 268 | 574 | 471 | 642 | 14 | 93 | 32 | 6 | 16 | 30 | 30 | 12 | 2188 | | 3 | | 38 | 41 | |
|  | Dla_WT_5_r1 | 345 | 619 | 469 | 647 | 11 | 65 | 20 | 6 | 4 | 25 | 12 | 5 | 2228 | | 1 | | 58 | 59 | |
|  | Dla_WT_5_r2 | 173 | 291 | 205 | 278 | 8 | 25 | 8 | 6 | 4 | 10 | 10 | 6 | 1024 | | 2 | | 41 | 43 | |
|  | Dla_WT_5_r3 | 180 | 304 | 217 | 384 | 4 | 25 | 11 | 11 | 5 | 8 | 12 | 7 | 1168 | | 2 | | 53 | 55 | |
|  | Dla_Mut_1_r1 | 122 | 268 | 270 | 172 | 6 | 41 | 18 | 3 | 2 | 16 | 24 | 11 | 953 | | 5 | | 9 | 14 | |
|  | Dla_Mut_1_r2 | 29 | 136 | 134 | 126 | 8 | 11 | 9 | 3 | 5 | 12 | 9 | 8 | 490 | | 3 | | 11 | 14 | |
|  | Dla_Mut_1_r3 | 74 | 221 | 238 | 115 | 10 | 24 | 11 | 3 | 1 | 10 | 23 | 9 | 739 | | 1 | | 3 | 4 | |
|  | Dla_Mut_5_r1 | 124 | 250 | 169 | 259 | 4 | 16 | 4 | 1 | 1 | 29 | 10 | 7 | 874 | | 3 | | 13 | 16 | |
|  | Dla_Mut_5_r2 | 107 | 240 | 165 | 234 | 1 | 22 | 8 | 4 | 1 | 19 | 11 | 7 | 819 | | 1 | | 9 | 10 | |
|  | Dla_Mut_5_r3 | 86 | 161 | 128 | 123 | 1 | 16 | 10 | 4 | 3 | 8 | 9 | 6 | 555 | | 0 | | 9 | 9 | |

**Supplementary Table 2.** Summary of mutations present in RNA2 quasispecies of RGNNV in sea bass.

| **Sample** | | **SNP** | | | | | | | | | | | | | **InDels** | | | |
| --- | --- | --- | --- | --- | --- | --- | --- | --- | --- | --- | --- | --- | --- | --- | --- | --- | --- | --- |
|  |  | **Transitions (Ts)** | | | | **Transversions (Tv)** | | | | | | | | **Total** | **Insertions** | | **Deletions** | **Total** |
|  |  | A → G | C → T | G → A | T → C | A → C | A → T | C → A | C → G | G → C | G → T | T → A | T → G |  |  |  | |  |
|  | Dla_WT_1_r1 | 257 | 377 | 319 | 299 | 17 | 46 | 8 | 1 | 2 | 15 | 3 | 1 | 1345 | 2 | 15 | | 17 |
|  | Dla_WT_1_r2 | 243 | 371 | 315 | 295 | 14 | 60 | 10 | 2 | 4 | 15 | 12 | 2 | 1343 | 1 | 27 | | 28 |
|  | Dla_WT_1_r3 | 225 | 373 | 296 | 300 | 20 | 67 | 8 | 1 | 11 | 16 | 9 | 0 | 1326 | 2 | 18 | | 20 |
|  | Dla_WT_5_r1 | 262 | 379 | 320 | 296 | 16 | 40 | 5 | 3 | 3 | 14 | 10 | 1 | 1349 | 0 | 24 | | 24 |
|  | Dla_WT_5_r2 | 261 | 374 | 316 | 297 | 14 | 33 | 4 | 2 | 6 | 8 | 10 | 0 | 1325 | 0 | 30 | | 30 |
|  | Dla_WT_5_r3 | 247 | 380 | 322 | 292 | 12 | 42 | 6 | 0 | 5 | 12 | 9 | 0 | 1327 | 0 | 32 | | 32 |
|  | Dla_Mut_1_r1 | 183 | 276 | 263 | 188 | 14 | 89 | 34 | 0 | 3 | 21 | 64 | 9 | 1144 | 2 | 14 | | 16 |
|  | Dla_Mut_1_r2 | 194 | 268 | 259 | 189 | 17 | 84 | 32 | 2 | 4 | 26 | 72 | 10 | 1157 | 5 | 16 | | 21 |
|  | Dla_Mut_1_r3 | 178 | 258 | 243 | 185 | 14 | 84 | 29 | 1 | 7 | 20 | 65 | 9 | 1093 | 1 | 19 | | 20 |
|  | Dla_Mut_5_r1 | 169 | 278 | 229 | 248 | 16 | 79 | 18 | 1 | 12 | 21 | 24 | 16 | 1111 | 3 | 14 | | 17 |
|  | Dla_Mut_5_r2 | 245 | 362 | 310 | 291 | 19 | 51 | 8 | 4 | 10 | 16 | 5 | 3 | 1324 | 1 | 34 | | 35 |
|  | Dla_Mut_5_r3 | 253 | 338 | 256 | 249 | 11 | 40 | 15 | 2 | 10 | 25 | 16 | 2 | 1217 | 1 | 42 | | 43 |

**Supplementary Table 3.** Summary of mutations present in RNA1 RGNNV in sea bream.

| **Sample** | | **SNP** | | | | | | | | | | | | | | **InDels** | | | | |
| --- | --- | --- | --- | --- | --- | --- | --- | --- | --- | --- | --- | --- | --- | --- | --- | --- | --- | --- | --- | --- |
|  |  | **Transitions (Ts)** | | | | **Transversions (Tv)** | | | | | | | | | **Total** | **Insertions** | | **Deletions** | | **Total** |
|  |  | A → G | C → T | G → A | T → C | A → C | A → T | C → A | C → G | G → C | G → T | T → A | T → G |  | |  |  | |  | |
|  | Sau_WT_1_r1 | 99 | 246 | 206 | 289 | 12 | 34 | 17 | 3 | 4 | 19 | 22 | 13 | 964 | | 5 | 19 | | 24 | |
|  | Sau_WT_1_r2 | 481 | 700 | 575 | 686 | 17 | 103 | 32 | 6 | 12 | 44 | 20 | 6 | 2682 | | 3 | 26 | | 29 | |
|  | Sau_WT_1_r3 | 51 | 152 | 115 | 191 | 7 | 13 | 11 | 3 | 8 | 8 | 15 | 11 | 585 | | 1 | 12 | | 13 | |
|  | Sau_WT_5_r1 | 331 | 375 | 299 | 368 | 8 | 37 | 34 | 12 | 6 | 37 | 18 | 19 | 1544 | | 5 | 64 | | 69 | |
|  | Sau_WT_5_r2 | 283 | 321 | 241 | 345 | 7 | 29 | 18 | 3 | 6 | 25 | 12 | 14 | 1304 | | 2 | 40 | | 42 | |
|  | Sau_WT_5_r3 | 234 | 337 | 269 | 326 | 7 | 52 | 20 | 10 | 6 | 15 | 26 | 17 | 1319 | | 4 | 23 | | 27 | |

**Supplementary Table 4.** Summary of mutations present in RNA2 quasispecies of RGNNV in sea bream.

| **Sample** | | **SNP** | | | | | | | | | | | | | | **InDels** | | | | |
| --- | --- | --- | --- | --- | --- | --- | --- | --- | --- | --- | --- | --- | --- | --- | --- | --- | --- | --- | --- | --- |
|  |  | **Transitions (Ts)** | | | | **Transversions (Tv)** | | | | | | | | | **Total** | **Insertions** | | **Deletions** | | **Total** |
|  |  | A → G | C → T | G → A | T → C | A → C | A → T | C → A | C → G | G → C | G → T | T → A | T → G |  | |  |  | |  | |
|  | Sau_WT_1_r1 | 265 | 345 | 279 | 288 | 13 | 42 | 16 | 3 | 8 | 28 | 14 | 5 | 1306 | | 2 | 28 | | 30 | |
|  | Sau_WT_1_r2 | 262 | 368 | 296 | 291 | 12 | 45 | 11 | 1 | 10 | 25 | 9 | 4 | 1334 | | 2 | 28 | | 30 | |
|  | Sau_WT_1_r3 | 260 | 331 | 283 | 277 | 12 | 43 | 16 | 2 | 6 | 21 | 13 | 11 | 1275 | | 2 | 22 | | 24 | |
|  | Sau_WT_5_r1 | 257 | 372 | 275 | 313 | 16 | 48 | 12 | 0 | 2 | 66 | 6 | 0 | 1367 | | 2 | 15 | | 17 | |
|  | Sau_WT_5_r2 | 249 | 376 | 301 | 302 | 17 | 52 | 8 | 0 | 5 | 30 | 6 | 2 | 1348 | | 2 | 18 | | 20 | |
|  | Sau_WT_5_r3 | 253 | 377 | 325 | 300 | 12 | 50 | 6 | 0 | 1 | 12 | 7 | 2 | 1345 | | 1 | 17 | | 18 | |
