# Supplementary material for: The genetic variability and evolution of red-spotted grouper nervous necrosis virus quasispecies can be associated with its virulence
Source: Front Microbiol. 2023 Jun 15;14:1182695. doi: 10.3389/fmicb.2023.1182695 (PMC10308047; doi:10.3389/fmicb.2023.1182695)
Supplement: Supplementary file 1 [file Data_Sheet_1.zip › Supplementary Material S5.docx]

Supplementary Material S5

**The genetic variability and evolution of red-spotted grouper nervous necrosis virus quasispecies can be associated with its virulence**

**Sergio Ortega-del Campo, Luis Díaz-Martínez, Patricia Moreno, Esther García-Rosado, M. Carmen Alonso, Julia Béjar* and Ana Grande-Pérez***

*** Correspondence:** Corresponding Author: bejar@uma.es & agrande@uma.es

**Supplementary Table 1.** Chi-square test analysis of substitution biases in the RNA1 segment of virulent red-spotted grouper nervous necrosis virus (RGNNV) quasispecies (rDl965), in sea bass at 1 and 5 dpi.

| **Genetic variation** | **RNA1_ Dla_WT_1** | | | | **RNA1_ Dla_WT_5** | | | |
| --- | --- | --- | --- | --- | --- | --- | --- | --- |
|  | **Obs.^a^** | **Exp.^b^** | **p-value^c^** | **%^d^** | **Obs.** | **Exp.** | **p-value** | **%** |
| **Transversions** | 701 | 4843.33 |  | 9.61 | 308 | 2946.67 |  | 6.73 |
| A → C | 53 | 565.70 | 1.26 × 10^-101^ | 0.73 | 23 | 344.17 | 8.30 × 10^-66^ | 0.50 |
| A → T | 326 | 565.70 | 8.81 × 10^-23^ | 4.47 | 115 | 344.17 | 7.31 × 10^-34^ | 2.51 |
| C → A | 98 | 661.60 | 5.55 × 10^-105^ | 1.34 | 39 | 402.51 | 5.15 × 10^-72^ | 0.85 |
| C → G | 12 | 661.60 | 3.16 × 10^-139^ | 0.16 | 23 | 402.51 | 1.99 × 10^-78^ | 0.50 |
| G → C | 29 | 608.56 | 1.40 × 10^-120^ | 0.40 | 13 | 370.25 | 1.41 × 10^-75^ | 0.28 |
| G → T | 86 | 608.56 | 3.65 × 10^-98^ | 1.18 | 43 | 370.25 | 1.56 × 10^-63^ | 0.94 |
| T → A | 63 | 585.80 | 4.83 × 10^-102^ | 0.86 | 34 | 356.40 | 4.68 × 10^-64^ | 0.74 |
| T → G | 34 | 585.80 | 1.36 × 10^-113^ | 0.47 | 18 | 356.40 | 1.69 × 10^-70^ | 0.39 |
| **Transitions** | 6564 | 2421.67 |  | 90.03 | 4112 | 1473.33 |  | 89.84 |
| A → G | 1020 | 565.70 | 5.99 × 10^-80^ | 13.99 | 698 | 344.17 | 1.03 × 10^-79^ | 15.25 |
| C → T | 1952 | 661.60 | <10^-200^ | 26.77 | 1214 | 402.51 | <10^-200^ | 26.52 |
| G → A | 1637 | 608.56 | <10^-200^ | 22.45 | 891 | 370.25 | <10^-200^ | 19.47 |
| T → C | 1955 | 585.80 | <10^-200^ | 26.81 | 1309 | 356.40 | <10^-200^ | 28.60 |
| **Total substitutions** | 7265 | 7265.00 |  | 99.64 | 4420 | 4420.00 |  | 96.57 |
| **Insertions** | 5 |  |  | 0.07 | 5 |  |  | 0.11 |
| **Deletions** | 21 |  |  | 0.29 | 152 |  |  | 3.32 |
| **Total** | 7291 |  |  | 100 | 4577 |  |  | 100 |
| **GC Content** |  |  |  |  |  |  |  |  |
| A/T→G/C | 3062 | 2303.01 |  | 42.15 | 2048 | 1401.14 |  | 46.33 |
| G/C→A/T | 3773 | 2540.33 |  | 51.93 | 2187 | 1545.53 |  | 49.48 |
| **Transitions (Ts) vs transversions (Tv)** | | |  |  |  |  |  |  |
| Ts | 6564 | 2421.67 |  | 90.35 | 4112 | 1473.33 |  | 93.03 |
| Tv | 701 | 4843.33 |  | 9.65 | 308 | 2946.67 |  | 6.97 |
| Ts/Tv | 9.36 | 0.50 |  |  | 13.35 | 0.50 |  |  |

^a^Observed mutations of each type.

^b^Expected mutations of each type.

^c^p-value of the chi^2^ statistical analysis.

^d^Proportion in % of observed mutations.

**Supplementary Table 2.** Chi-square test analysis of substitution biases in the RNA1 segment of low virulent RGNNV quasispecies (Mut270Dl965), in sea bass at 1 and 5 dpi.

| **Genetic variation** | **RNA1_ Dla_Mut_1** | | | | **RNA1_ Dla_Mut_5** | | | |
| --- | --- | --- | --- | --- | --- | --- | --- | --- |
|  | **Obs.^a^** | **Exp.^b^** | **p-value^c^** | **%^d^** | **Obs.** | **Exp.** | **p-value** | **%** |
| **Transversions** | 277 | 1454.67 |  | 12.51 | 202 | 1498.67 |  | 8.85 |
| A → C | 24 | 169.91 | 6.20 × 10^-28^ | 1.08 | 6 | 175.04 | 3.55 × 10^-36^ | 0.26 |
| A → T | 76 | 169.91 | 5.37 × 10^-12^ | 3.43 | 54 | 175.04 | 6.67 × 10^-19^ | 2.37 |
| C → A | 38 | 198.71 | 5.98 × 10^-29^ | 1.72 | 22 | 204.72 | 3.87 × 10^-36^ | 0.96 |
| C → G | 9 | 198.71 | 4.69 × 10^-40^ | 0.41 | 9 | 204.72 | 2.34 × 10^-41^ | 0.39 |
| G → C | 8 | 182.78 | 5.11 × 10^-37^ | 0.36 | 5 | 188.31 | 1.79 × 10^-39^ | 0.22 |
| G → T | 38 | 182.78 | 1.25 × 10^-25^ | 1.72 | 56 | 188.31 | 6.51 × 10^-21^ | 2.45 |
| T → A | 56 | 175.94 | 1.76 × 10^-18^ | 2.53 | 30 | 181.26 | 3.89 × 10^-28^ | 1.31 |
| T → G | 28 | 175.94 | 9.71 × 10^-28^ | 1.26 | 20 | 181.26 | 7.01 × 10^-32^ | 0.88 |
| **Transitions** | 1905 | 727.33 |  | 86.04 | 2046 | 749.33 |  | 89.62 |
| A → G | 225 | 169.91 | 1.32 × 10^-4^ | 10.16 | 317 | 175.04 | 1.00 × 10^-25^ | 13.89 |
| C → T | 625 | 198.71 | <10^-200^ | 28.23 | 651 | 204.72 | <10^-200^ | 28.52 |
| G → A | 642 | 182.78 | <10^-200^ | 29.00 | 462 | 188.31 | <10^-200^ | 20.24 |
| T → C | 413 | 175.94 | <10^-200^ | 18.65 | 616 | 181.26 | 4.17 × 10^-87^ | 26.98 |
| **Total substitutions** | 2182 | 2182.00 |  | 98.55 | 2248 | 2248.00 |  | 98.47 |
| **Insertions** | 9 |  |  | 0.41 | 4 |  |  | 0.18 |
| **Deletions** | 23 |  |  | 1.04 | 31 |  |  | 1.36 |
| **Total** | 2214 |  |  | 100 | 2283 |  |  | 100 |
| **GC Content** |  |  |  |  |  |  |  |  |
| A/T→G/C | 690 | 691.69 |  | 31.62 | 959 | 712.62 |  | 42.66 |
| G/C→A/T | 1343 | 762.97 |  | 61.55 | 1191 | 786.05 |  | 52.98 |
| **Transitions (Ts) vs transversions (Tv)** | |  |  |  |  |  |  |  |
| Ts | 1905 | 727.33 |  | 87.31 | 2046 | 749.33 |  | 91.01 |
| Tv | 277 | 1454.67 |  | 12.69 | 202 | 1498.67 |  | 8.99 |
| Ts/Tv | 6.88 | 0.50 |  |  | 10.13 | 0.50 |  |  |

^a^Observed mutations of each type.

^b^Expected mutations of each type.

^c^p-value of the chi^2^ statistical analysis.

^d^Proportion in % of observed mutations.

**Supplementary Table 3.** Chi-square test analysis of substitution biases in the RNA1 segment of RGNNV rDl965 quasispecies in sea bream at 1 and 5 dpi.

| **Genetic variation** | **RNA1_ Sau_WT_1** | | | | **RNA1_ Sau_WT_5** | | | |
| --- | --- | --- | --- | --- | --- | --- | --- | --- |
|  | **Obs.^a^** | **Exp.^b^** | **p-value^c^** | **%^d^** | **Obs.** | **Exp.** | **p-value** | **%** |
| **Transversions** | 440 | 2820.67 |  | 10.24 | 438 | 2778.00 |  | 10.17 |
| A → C | 36 | 329.45 | 1.74 × 10^-57^ | 0.84 | 22 | 324.47 | 5.93 × 10^-62^ | 0.51 |
| A → T | 150 | 329.45 | 5.95 × 10^-22^ | 3.49 | 118 | 324.47 | 2.95 × 10^-29^ | 2.74 |
| C → A | 60 | 385.30 | 2.30 × 10^-60^ | 1.40 | 72 | 379.47 | 7.96 × 10^-55^ | 1.67 |
| C → G | 12 | 385.30 | 2.90 × 10^-79^ | 0.28 | 25 | 379.47 | 1.25 × 10^-72^ | 0.58 |
| G → C | 24 | 354.42 | 1.29 × 10^-67^ | 0.56 | 18 | 349.06 | 6.60 × 10^-69^ | 0.42 |
| G → T | 71 | 354.42 | 6.11 × 10^-50^ | 1.65 | 77 | 349.06 | 9.03 × 10^-47^ | 1.79 |
| T → A | 57 | 341.16 | 4.03 × 10^-52^ | 1.33 | 56 | 336.00 | 2.15 × 10^-51^ | 1.30 |
| T → G | 30 | 341.16 | 2.37 × 10^-62^ | 0.70 | 50 | 336.00 | 1.37 × 10^-53^ | 1.16 |
| **Transitions** | 3791 | 1410.33 |  | 88.22 | 3729 | 1389.00 |  | 86.62 |
| A → G | 631 | 329.45 | 1.17 × 10^-60^ | 14.68 | 848 | 324.47 | 3.75 × 10^-184^ | 19.70 |
| C → T | 1098 | 385.30 | <10^-200^ | 25.55 | 1033 | 379.47 | <10^-200^ | 24.00 |
| G → A | 896 | 354.42 | <10^-200^ | 20.85 | 809 | 349.06 | <10^-200^ | 18.79 |
| T → C | 1166 | 341.16 | 1.95 × 10^-180^ | 27.14 | 1039 | 336.00 | 2.49 × 10^-132^ | 24.13 |
| **Total substitutions** | 4231 | 4231.00 |  | 98.46 | 4167 | 4167.00 |  | 96.79 |
| **Insertions** | 9 |  |  | 0.21 | 11 |  |  | 0.26 |
| **Deletions** | 57 |  |  | 1.33 | 127 |  |  | 2.95 |
| **Total** | 4297 |  |  | 100 | 4305 |  |  | 100 |
| **GC Content** |  |  |  |  |  |  |  |  |
| A/T→G/C | 1863 | 1341.23 |  | 44.03 | 1959 | 1320.94 |  | 47.01 |
| G/C→A/T | 2125 | 1479.44 |  | 50.22 | 1991 | 1457.06 |  | 47.78 |
| **Transitions (Ts) vs transversions (Tv)** | |  |  |  |  |  |  |  |
| Ts | 3791 | 1410.33 |  | 89.60 | 3729 | 1389.00 |  | 89.49 |
| Tv | 440 | 2820.67 |  | 10.40 | 438 | 2778.00 |  | 10.51 |
| Ts/Tv | 8.62 | 0.50 |  |  | 8.51 | 0.50 |  |  |

^a^Observed mutations of each type.

^b^Expected mutations of each type.

^c^p-value of the chi^2^ statistical analysis.

^d^Proportion in % of observed mutations.

**Supplementary Table 4.** Chi-square test analysis of substitution biases in the RNA2 segment of virulent RGNNV quasispecies (rDl965), in sea bass at 1 and 5 dpi.

| **Genetic variation** | **RNA2_ Dla_WT_1** | | | | **RNA2_ Dla_WT_5** | | | |
| --- | --- | --- | --- | --- | --- | --- | --- | --- |
|  | **Obs.^a^** | **Exp.^b^** | **p-value^c^** | **%^d^** | **Obs.** | **Exp.** | **p-value** | **%** |
| **Transversions** | 344 | 2676.00 |  | 8.48 | 255 | 2667.33 |  | 6.24 |
| A → C | 51 | 327.94 | 1.64 × 10^-51^ | 1.26 | 42 | 326.88 | 1.22 × 10^-54^ | 1.03 |
| A → T | 173 | 327.94 | 1.27 × 10^-16^ | 4.26 | 115 | 326.88 | 1.50 × 10^-30^ | 2.81 |
| C → A | 26 | 376.51 | 1.39 × 10^-71^ | 0.64 | 15 | 375.29 | 7.77 × 10^-76^ | 0.37 |
| C → G | 4 | 376.51 | 9.31 × 10^-81^ | 0.10 | 5 | 375.29 | 4.60 × 10^-80^ | 0.12 |
| G → C | 17 | 334.50 | 3.63 × 10^-66^ | 0.42 | 14 | 333.42 | 3.56 × 10^-67^ | 0.34 |
| G → T | 46 | 334.50 | 9.29 × 10^-55^ | 1.13 | 34 | 333.42 | 4.10 × 10^-59^ | 0.83 |
| T → A | 24 | 299.04 | 1.17 × 10^-55^ | 0.59 | 29 | 298.07 | 1.80 × 10^-53^ | 0.71 |
| T → G | 3 | 299.04 | 2.29 × 10^-64^ | 0.07 | 1 | 298.07 | 5.10 × 10^-65^ | 0.02 |
| **Transitions** | 3670 | 1338.00 |  | 90.46 | 3746 | 1333.67 |  | 91.66 |
| A → G | 725 | 327.94 | 4.07E-105 | 17.87 | 770 | 326.88 | 3.65 × 10^-131^ | 18.84 |
| C → T | 1121 | 376.51 | <10^-200^ | 27.63 | 1133 | 375.29 | <10^-200^ | 27.72 |
| G → A | 930 | 334.50 | <10^-200^ | 22.92 | 958 | 333.42 | <10^-200^ | 23.44 |
| T → C | 894 | 299.04 | <10^-200^ | 22.04 | 885 | 298.07 | <10^-200^ | 21.65 |
| **Total substitutions** | 4014 | 4014.00 |  | 98.94 | 4001 | 4001.00 |  | 97.90 |
| **Insertions** | 1 |  |  | 0.02 | 0 |  |  | 0.00 |
| **Deletions** | 42 |  |  | 1.04 | 86 |  |  | 2.10 |
| **Total** | 4057 |  |  | 100 | 4087 |  |  | 100 |
| **GC Content** |  |  |  |  |  |  |  |  |
| A/T→G/C | 1673 | 1253.97 |  | 41.68 | 1698 | 1249.91 |  | 42.44 |
| G/C→A/T | 2123 | 1422.03 |  | 52.89 | 2140 | 1417.42 |  | 53.49 |
| **Transitions (Ts) vs transversions (Tv)** | |  |  |  |  |  |  |  |
| Ts | 3670 | 1338.00 |  | 91.43 | 3746 | 1333.67 |  | 93.63 |
| Tv | 344 | 2676.00 |  | 8.57 | 255 | 2667.33 |  | 6.37 |
| Ts/Tv | 10.67 | 0.50 |  |  | 14.69 | 0.50 |  |  |

^a^Observed mutations of each type.

^b^Expected mutations of each type.

^c^p-value of the chi^2^ statistical analysis.

^d^Proportion in % of observed mutations.

**Supplementary Table 5.** Chi-square test analysis of substitution biases in the RNA2 segment of low virulent RGNNV quasispecies (Mut270Dl965), in sea bass at 1 and 5 dpi.

| **Genetic variation** | **RNA2_ Dla_Mut_1** | | | | **RNA2_ Dla_Mut_5** | | | |
| --- | --- | --- | --- | --- | --- | --- | --- | --- |
|  | **Obs.^a^** | **Exp.^b^** | **p-value^c^** | **%^d^** | **Obs.** | **Exp.** | **p-value** | **%** |
| **Transversions** | 710 | 2262.67 |  | 20.57 | 424 | 2434.67 |  | 11.32 |
| A → C | 45 | 277.29 | 5.56 × 10^-43^ | 1.30 | 46 | 298.37 | 4.44 × 10^-47^ | 1.23 |
| A → T | 257 | 277.29 | 4.76 × 10^-1^ | 7.45 | 170 | 298.37 | 1.02 × 10^-12^ | 4.54 |
| C → A | 95 | 318.36 | 9.37 × 10^-35^ | 2.75 | 41 | 342.56 | 2.27 × 10^-58^ | 1.09 |
| C → G | 3 | 318.36 | 1.47 × 10^-68^ | 0.09 | 7 | 342.56 | 4.20 × 10^-72^ | 0.19 |
| G → C | 14 | 282.83 | 3.26 × 10^-56^ | 0.41 | 32 | 304.33 | 1.21 × 10^-36^ | 0.85 |
| G → T | 67 | 282.83 | 1.72 × 10^-36^ | 1.94 | 62 | 304.33 | 1.25 × 10^-42^ | 1.65 |
| T → A | 201 | 252.85 | 4.91 × 10^-3^ | 5.82 | 45 | 272.07 | 7.03 × 10^-42^ | 1.20 |
| T → G | 28 | 252.85 | 3.81 × 10^-44^ | 0.81 | 21 | 272.07 | 4.88 × 10^-51^ | 0.56 |
| **Transitions** | 2684 | 1131.33 |  | 77.77 | 3228 | 1217.33 |  | 86.15 |
| A → G | 555 | 277.29 | 4.02 × 10^-61^ | 16.08 | 667 | 298.37 | 1.27 × 10^-99^ | 17.80 |
| C → T | 802 | 318.36 | 2.84 × 10^-160^ | 23.24 | 978 | 342.56 | <10^-200^ | 26.10 |
| G → A | 765 | 282.83 | 3.22 × 10^-179^ | 22.17 | 795 | 304.33 | 1.65 × 10^-172^ | 21.22 |
| T → C | 562 | 252.85 | 8.39 × 10^-83^ | 16.29 | 788 | 272.07 | <10^-200^ | 21.03 |
| **Total substitutions** | 3394 | 3394.00 |  | 98.35 | 3652 | 3652.00 |  | 97.46 |
| **Insertions** | 8 |  |  | 0.23 | 5 |  |  | 0.13 |
| **Deletions** | 49 |  |  | 1.42 | 90 |  |  | 2.40 |
| **Total** | 3451 |  |  | 100 | 3747 |  |  | 100 |
| **GC Content** |  |  |  |  |  |  |  |  |
| A/T→G/C | 1190 | 1060.29 |  | 35.06 | 1522 | 1140.88 |  | 41.68 |
| G/C→A/T | 1729 | 1202.38 |  | 50.94 | 1876 | 1293.78 |  | 51.37 |
| **Transitions (Ts) vs transversions (Tv)** | |  |  |  |  |  |  |  |
| Ts | 2684 | 1131.33 |  | 79.08 | 3228 | 1217.33 |  | 88.39 |
| Tv | 710 | 2262.67 |  | 20.92 | 424 | 2434.67 |  | 11.61 |
| Ts/Tv | 3.78 | 0.50 |  |  | 7.61 | 0.50 |  |  |

^a^Observed mutations of each type.

^b^Expected mutations of each type.

^c^p-value of the chi^2^ statistical analysis.

^d^Proportion in % of observed mutations.

**Supplementary Table 6.** Chi-square test analysis for the detection of substitution biases in the RNA2 segment of rDl965 quasispecies in sea bream at 1 and 5 dpi.

| **Genetic variation** | **RNA2_ Sau_WT_1** | | | | **RNA2_ Sau_WT_5** | | | |
| --- | --- | --- | --- | --- | --- | --- | --- | --- |
|  | **Obs.^a^** | **Exp.^b^** | **p-value^c^** | **%^d^** | **Obs.** | **Exp.** | **p-value** | **%** |
| **Transversions** | 370 | 2610.00 |  | 9.25 | 360 | 2706.67 |  | 8.75 |
| A → C | 37 | 319.86 | 4.83 × 10^-55^ | 0.93 | 45 | 331.70 | 1.55 × 10^-54^ | 1.09 |
| A → T | 130 | 319.86 | 3.38 × 10^-25^ | 3.25 | 150 | 331.70 | 2.44E × 10^-22^ | 3.65 |
| C → A | 43 | 367.23 | 6.90 × 10^-63^ | 1.08 | 26 | 380.83 | 1.62 × 10^-72^ | 0.63 |
| C → G | 6 | 367.23 | 6.95 × 10^-78^ | 0.15 | 0 | 380.83 | 2.02 × 10^-83^ | 0.00 |
| G → C | 24 | 326.25 | 1.57 × 10^-61^ | 0.60 | 8 | 338.33 | 9.23 × 10^-71^ | 0.19 |
| G → T | 74 | 326.25 | 4.45 × 10^-43^ | 1.85 | 108 | 338.33 | 8.90 × 10^-35^ | 2.62 |
| T → A | 36 | 291.67 | 2.16 × 10^-49^ | 0.90 | 19 | 302.47 | 2.05 × 10^-58^ | 0.46 |
| T → G | 20 | 291.67 | 1.13 × 10^-55^ | 0.50 | 4 | 302.47 | 1.11 × 10^-64^ | 0.10 |
| **Transitions** | 3545 | 1305.00 |  | 88.65 | 3700 | 1353.33 |  | 89.91 |
| A → G | 787 | 319.86 | 7.07 × 10^-149^ | 19.68 | 759 | 331.70 | 2.97 × 10^-120^ | 18.44 |
| C → T | 1044 | 367.23 | <10^-200^ | 26.11 | 1125 | 380.83 | <10^-200^ | 27.34 |
| G → A | 858 | 326.25 | 6.32 × 10^-189^ | 21.46 | 901 | 338.33 | <10^-200^ | 21.90 |
| T → C | 856 | 291.67 | <10^-200^ | 21.41 | 915 | 302.47 | <10^-200^ | 22.24 |
| **Total substitutions** | 3915 | 3915.00 |  | 97.90 | 4060 | 4060.00 |  | 98.66 |
| **Insertions** | 6 |  |  | 0.15 | 5 |  |  | 0.12 |
| **Deletions** | 78 |  |  | 1.95 | 50 |  |  | 1.22 |
| **Total** | 3999 |  |  | 100 | 4115 |  |  | 100 |
| **GC Content** |  |  |  |  |  |  |  |  |
| A/T→G/C | 1700 | 1223.05 |  | 43.42 | 1723 | 1268.34 |  | 41.35 |
| G/C→A/T | 2019 | 1386.95 |  | 51.57 | 2160 | 1438.32 |  | 51.84 |
| **Transitions (Ts) vs transversions (Tv)** | |  |  |  |  |  |  |  |
| Ts | 3545 | 1305.00 |  | 90.55 | 3700 | 1353.33 |  | 88.79 |
| Tv | 370 | 2610.00 |  | 9.45 | 360 | 2706.67 |  | 8.64 |
| Ts/Tv | 9.58 | 0.50 |  |  | 10.28 | 0.50 |  |  |

^a^Observed mutations of each type.

^b^Expected mutations of each type.

^c^p-value of the chi^2^ statistical analysis.

^d^Proportion in % of observed mutations.
