# Supplementary material for: The genetic variability and evolution of red-spotted grouper nervous necrosis virus quasispecies can be associated with its virulence
Source: Front Microbiol. 2023 Jun 15;14:1182695. doi: 10.3389/fmicb.2023.1182695 (PMC10308047; doi:10.3389/fmicb.2023.1182695)
Supplement: Supplementary file 1 [file Data_Sheet_1.zip › Supplementary Material S6.docx]

Supplementary Material S6

**The genetic variability and evolution of red-spotted grouper nervous necrosis virus quasispecies can be associated with its virulence**

**Sergio Ortega-Del-Campo, Luis Díaz-Martínez, Patricia Moreno, Esther García-Rosado, M Carmen Alonso, Julia Béjar* and Ana Grande-Pérez***

*** Correspondence:** Corresponding Author: bejar@uma.es & agrande@uma.es


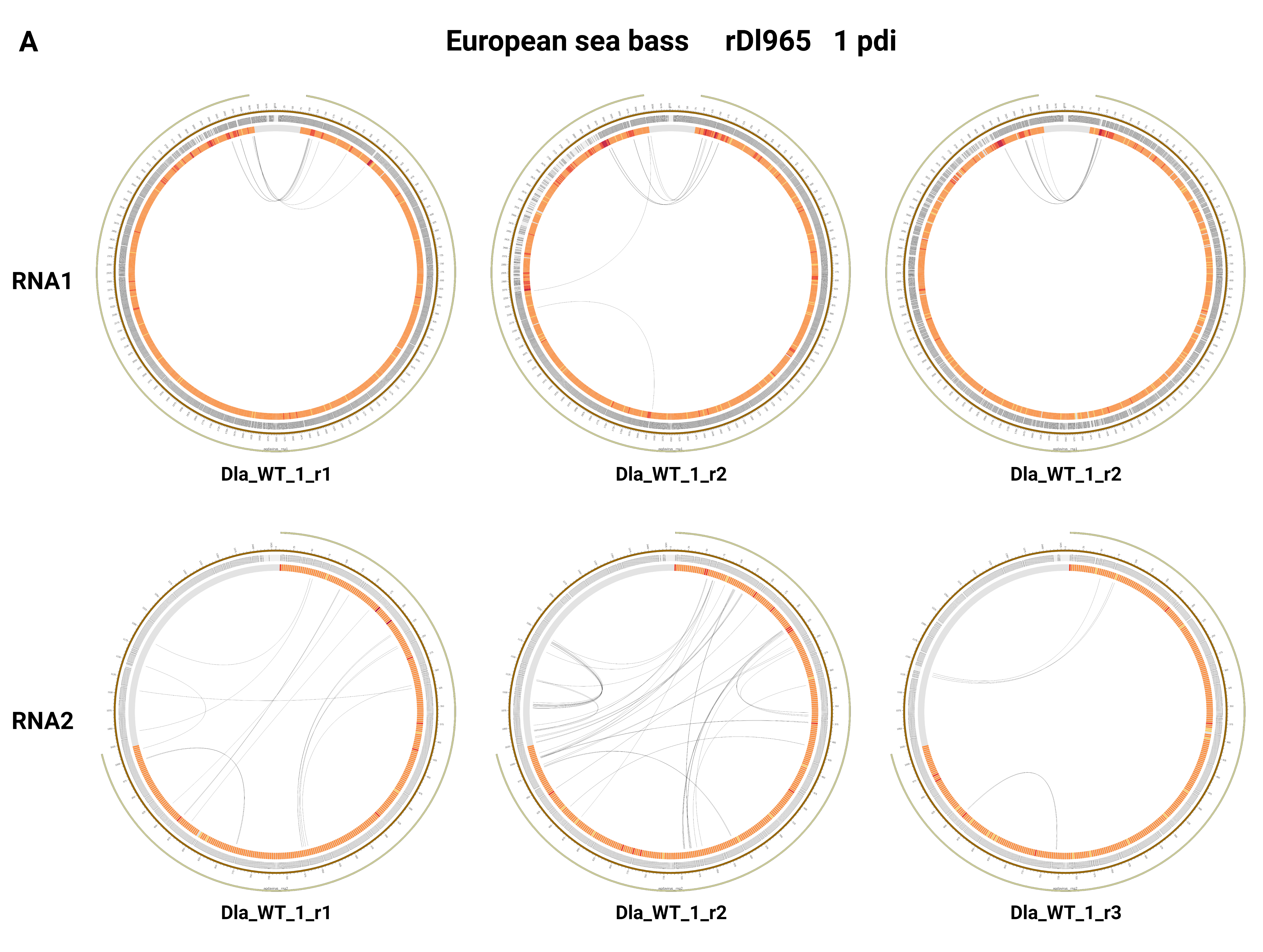


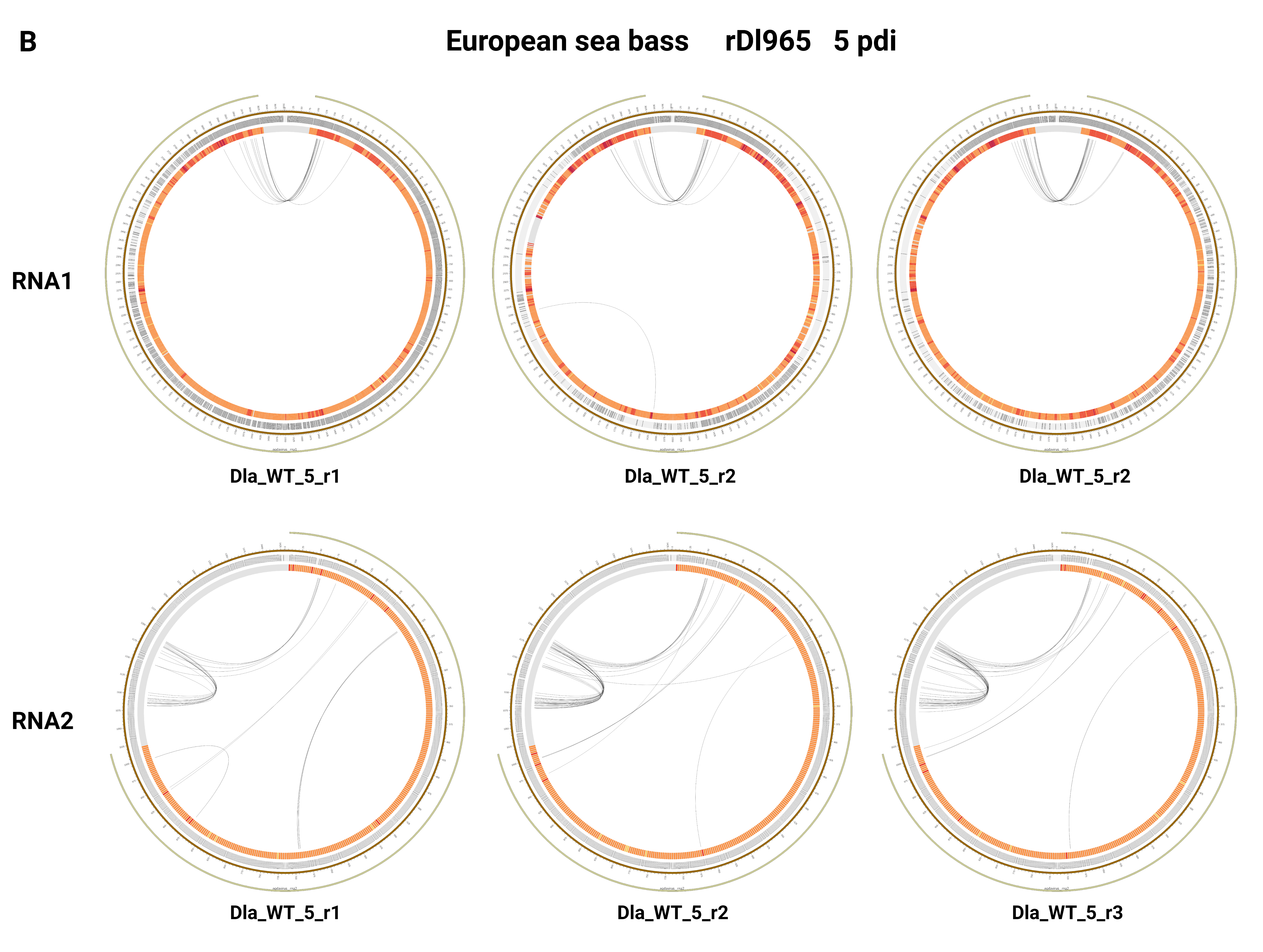


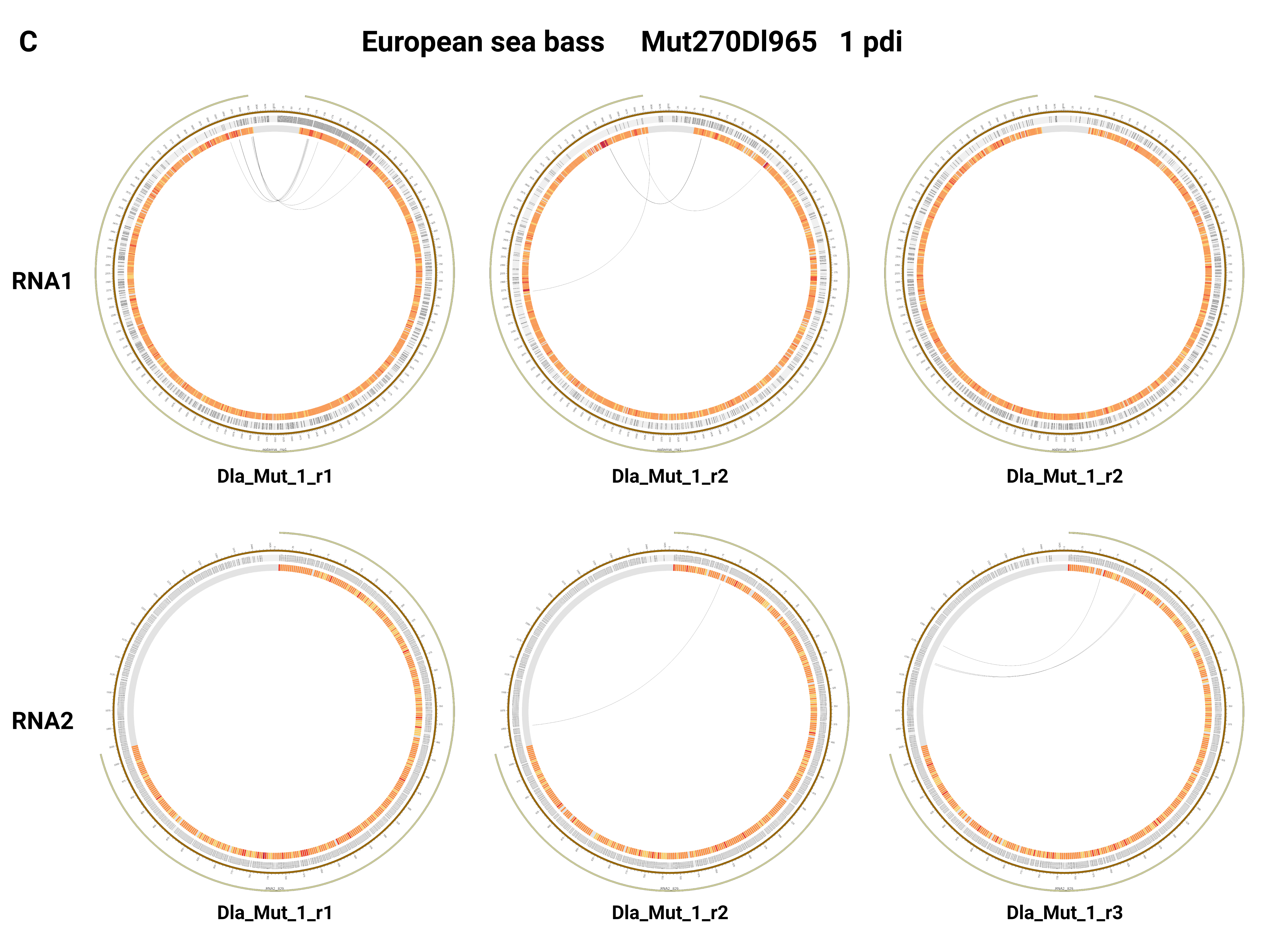


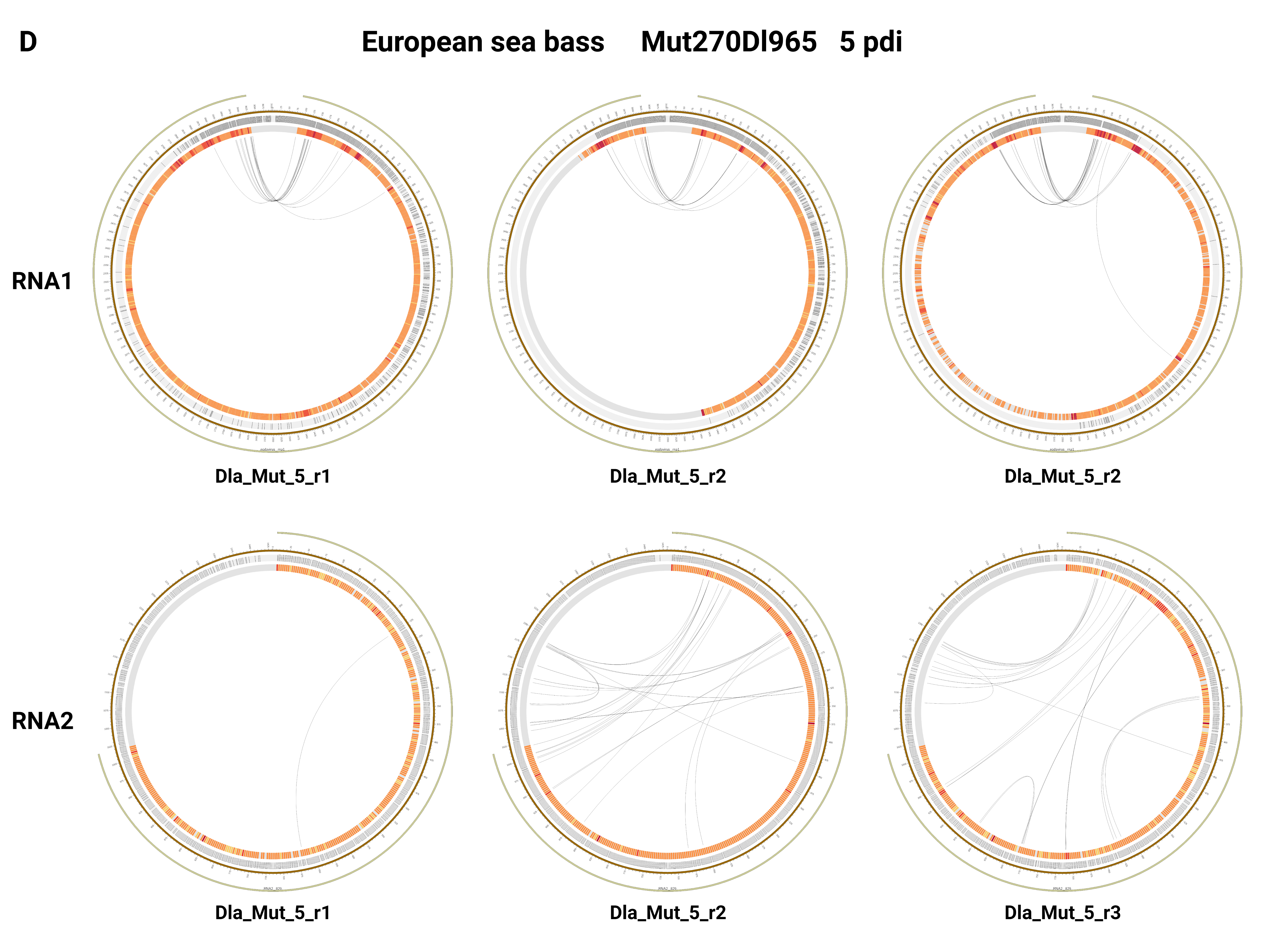


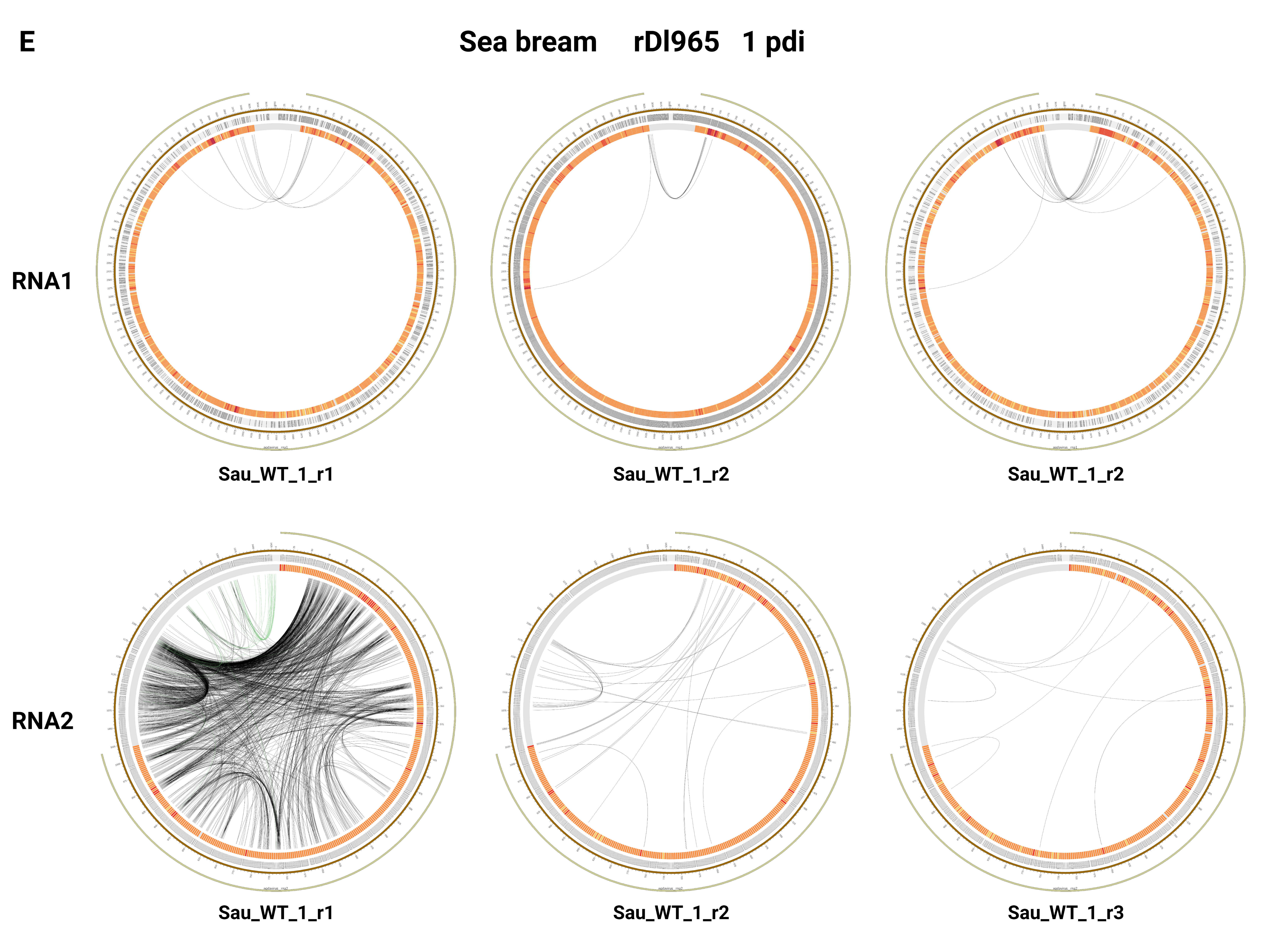


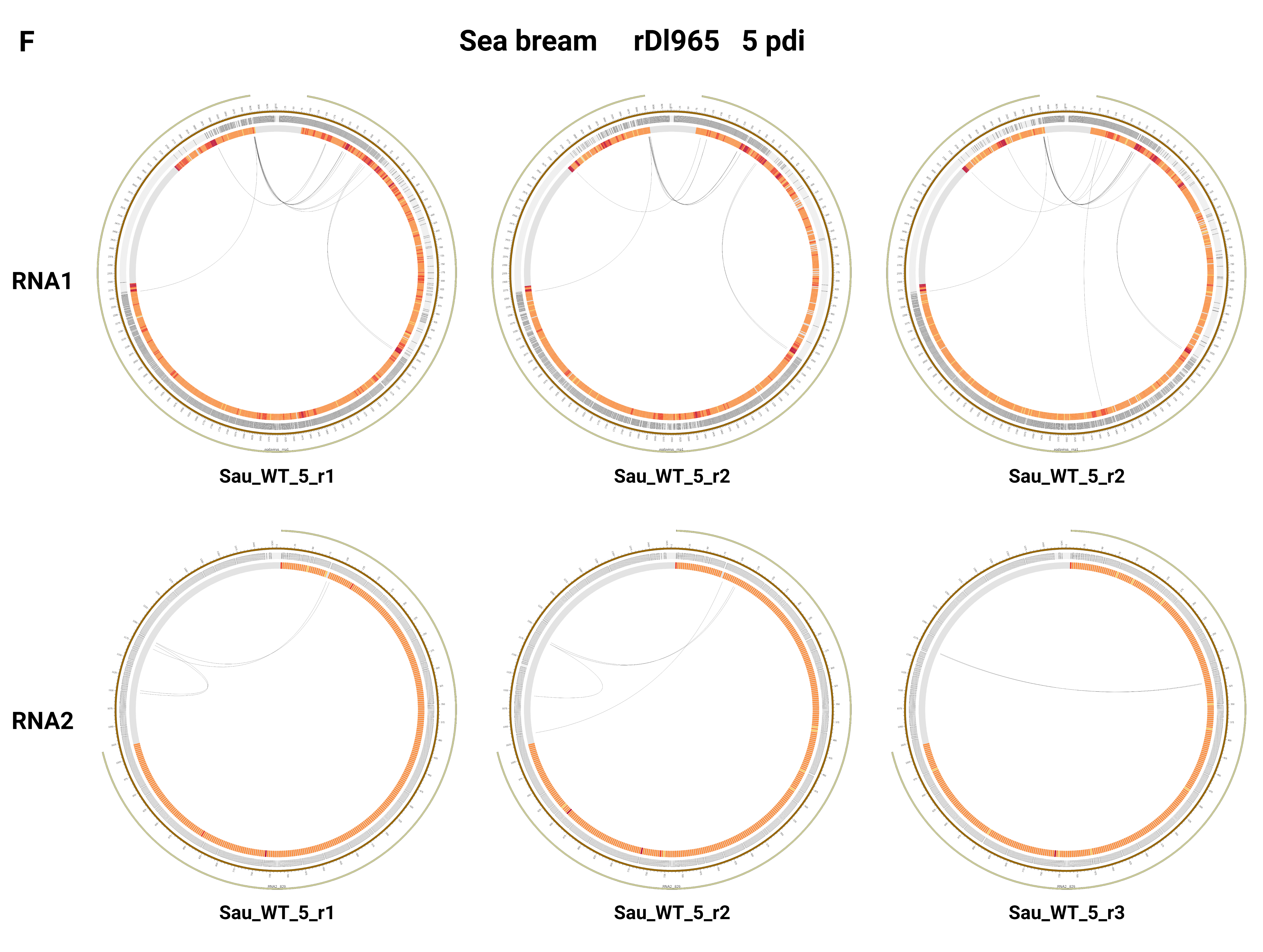


**Supplementary Figure 1.** Graphical representation of the mutation profile of red-spotted grouper nervous necrosis virus (RGNNV) quasispecies in both segments. The mutation frequency of nucleotides and codons is expressed by a color code from yellow to red. Recombination points have been represented by black lines. Each panel represents the results of the samples organized according to host, virus type and day of extraction: (A) rDl965 quasispecies extracted from sea bass at 1 dpi; (B) rDl965 quasispecies extracted from sea bass at 5 dpi, (C) Mut270Dl965 quasispecies extracted from sea bass at 1 dpi; (D) Mut270Dl965 quasispecies extracted from sea bass at 5 dpi; (E) rDl965 quasispecies extracted from sea bream at 1 dpi; (F) rDl965 quasispecies extracted from sea bream at 5 dpi.
