# Supplementary material for: The genetic variability and evolution of red-spotted grouper nervous necrosis virus quasispecies can be associated with its virulence
Source: Front Microbiol. 2023 Jun 15;14:1182695. doi: 10.3389/fmicb.2023.1182695 (PMC10308047; doi:10.3389/fmicb.2023.1182695)
Supplement: Supplementary file 1 [file Data_Sheet_1.zip › Supplementary Material S7.docx]

Supplementary Material S7

**The genetic variability and evolution of red-spotted grouper nervous necrosis virus quasispecies can be associated with its virulence**

**Sergio Ortega-del Campo, Luis Díaz-Martínez, Patricia Moreno, Esther García-Rosado, M. Carmen Alonso, Julia Béjar* and Ana Grande-Pérez***

*** Correspondence:** Corresponding Author: bejar@uma.es & agrande@uma.es


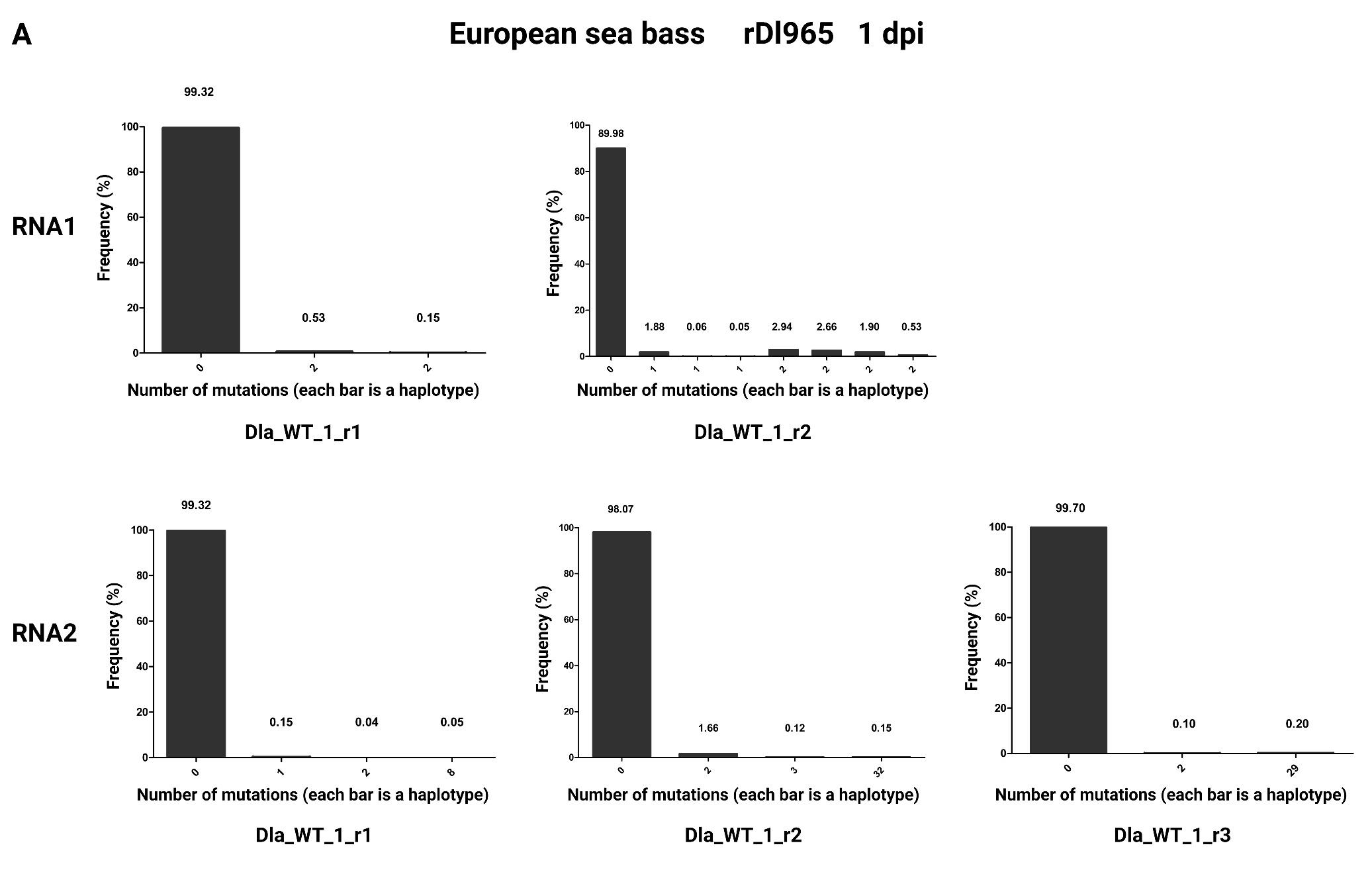


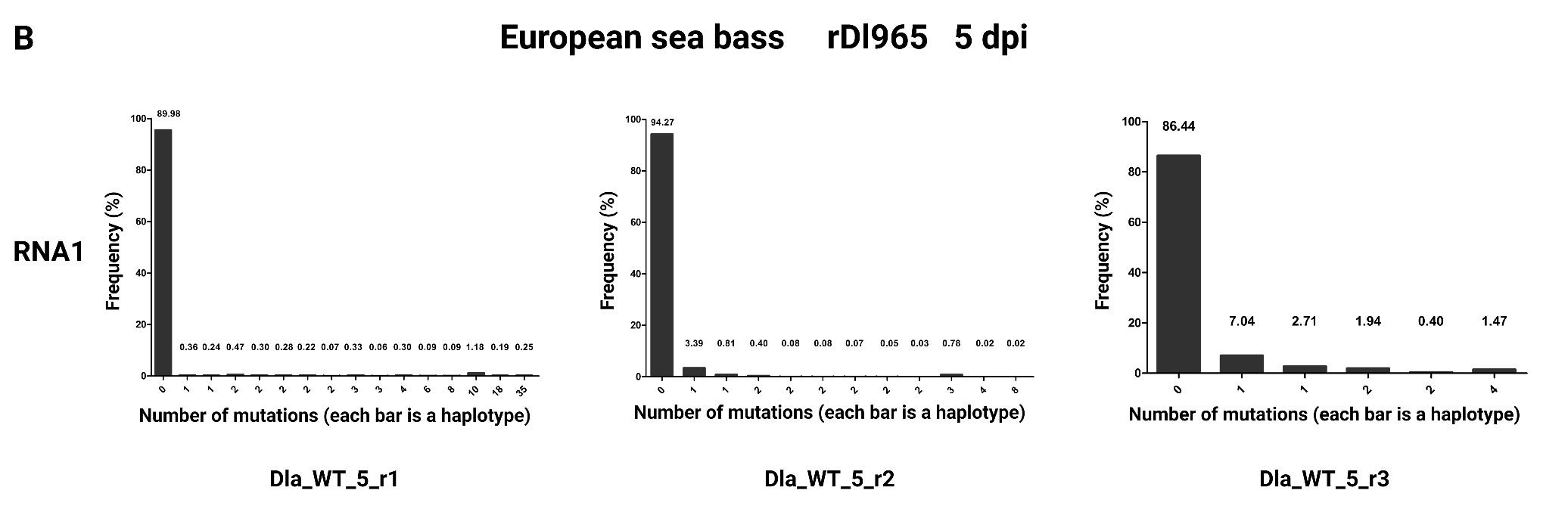


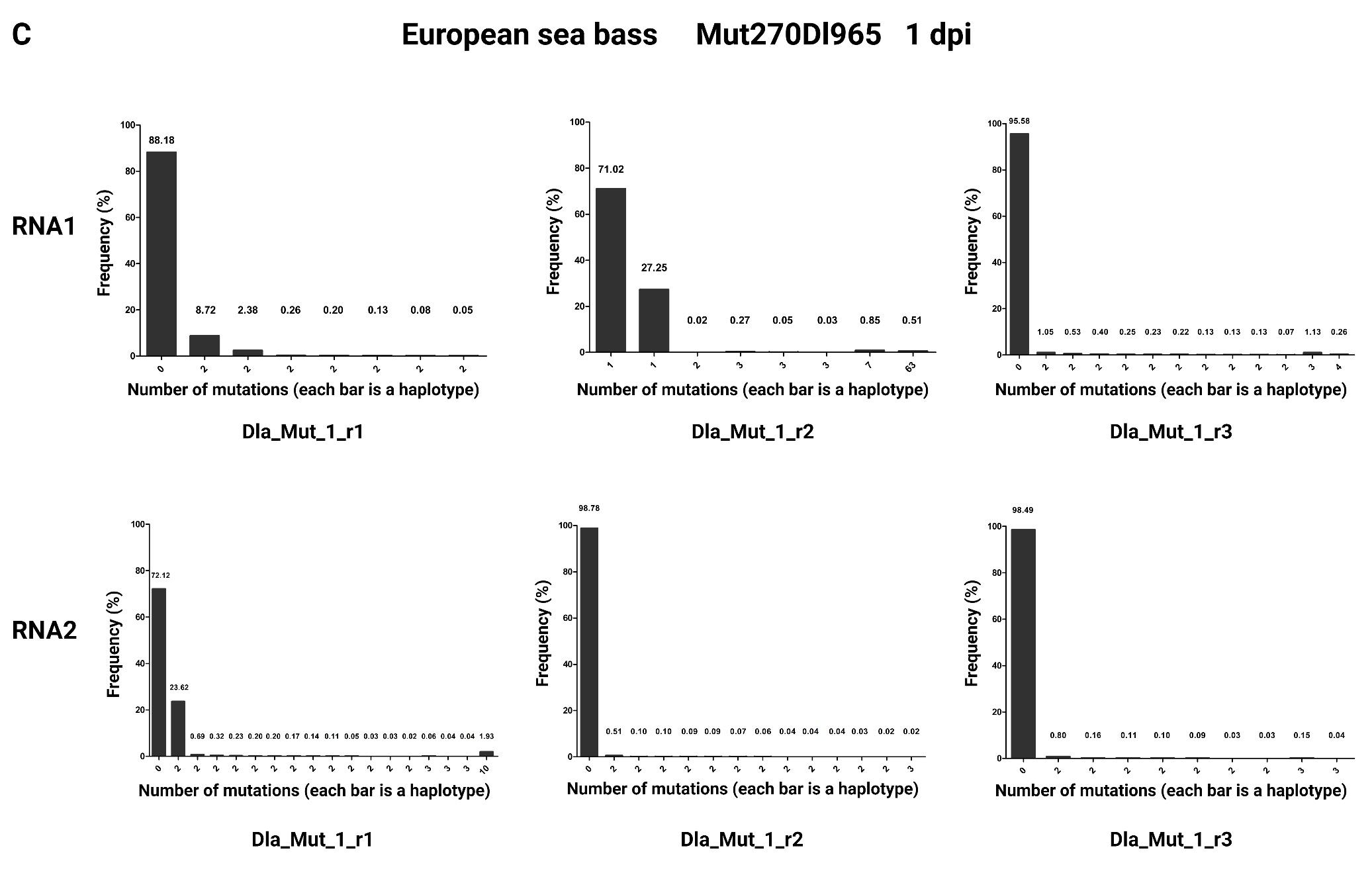


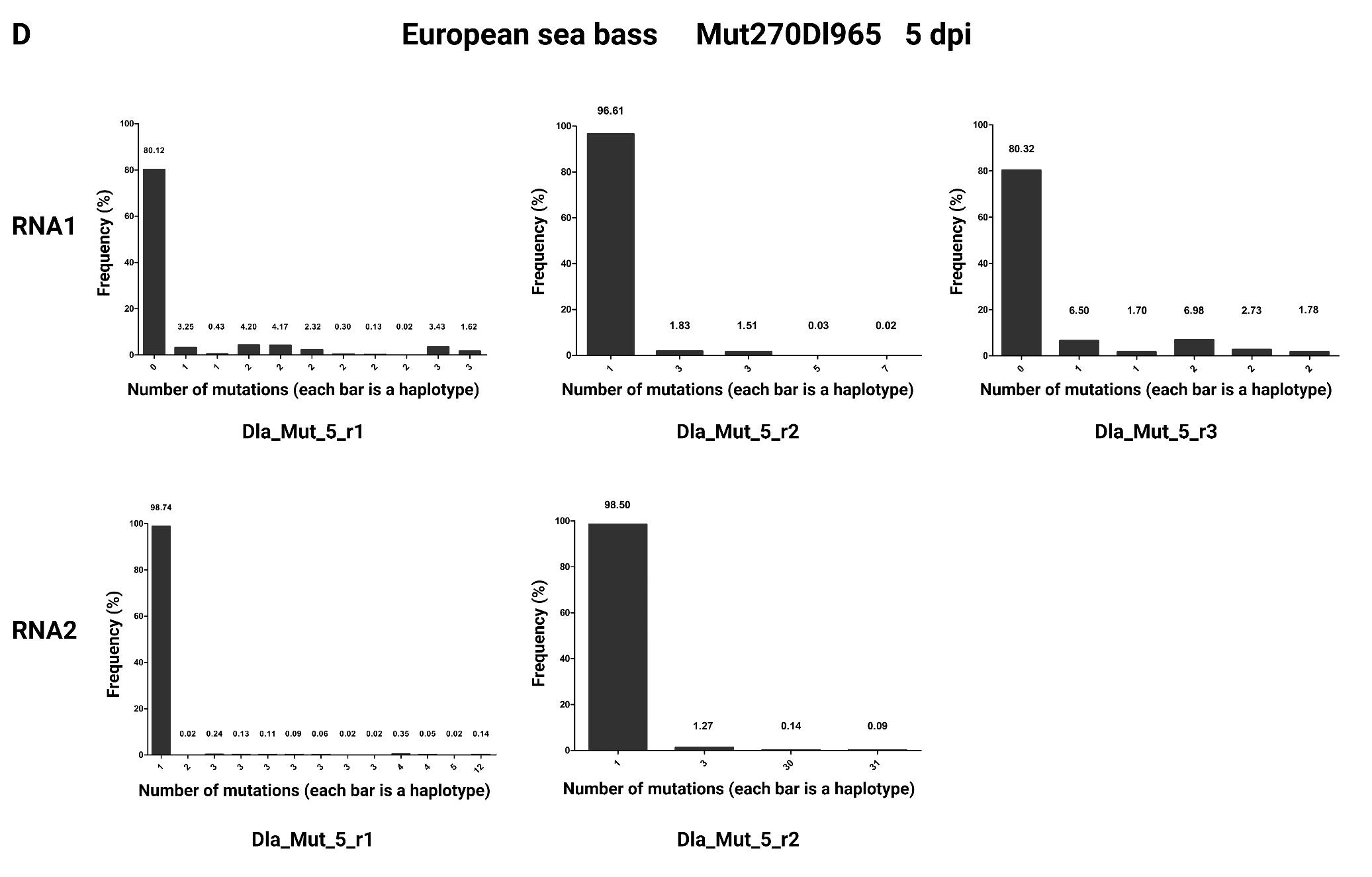


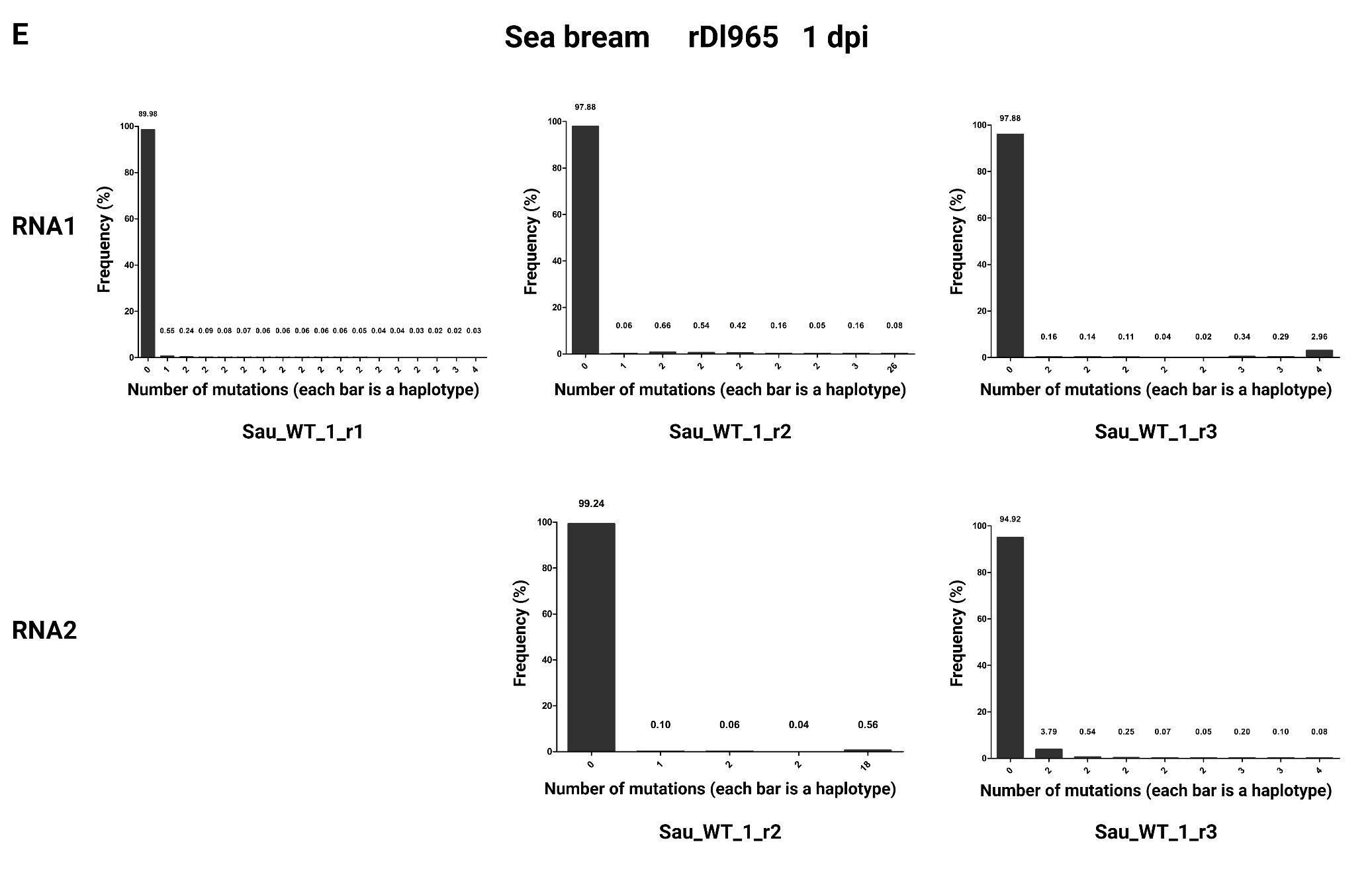


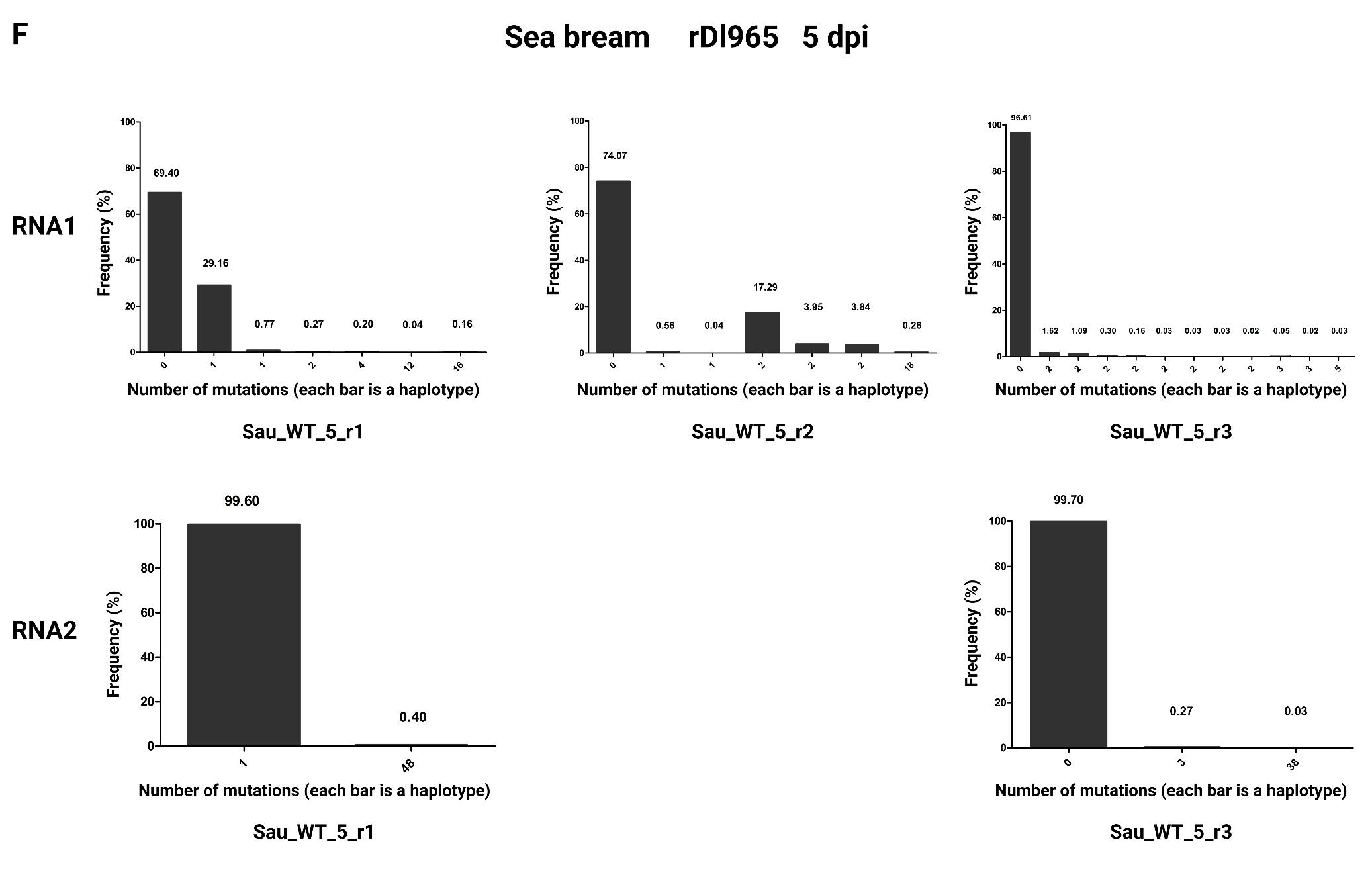


**Supplementary Figure 1.** Montserrat plots profiling the heterogeneity of red-spotted grouper nervous necrosis virus (RGNNV) quasispecies, indicating the number and weight of haplotypes in each sample. Each panel represents the sample results organized according to host, virus and day of extraction: (A) rDl965 quasispecies extracted from sea bass at 1 dpi, (B) rDl965 quasispecies extracted from sea bass at 5 dpi, (C) Mut270Dl965 quasispecies extracted from sea bass at 1 dpi, (D) Mut270Dl965 quasispecies extracted from sea bass at 5 dpi, (E) rDl965 quasispecies extracted from sea bream at 1 dpi, (F) rDl965 quasispecies extracted from sea bream at 5 dpi.
