# Supplementary material for: The genetic variability and evolution of red-spotted grouper nervous necrosis virus quasispecies can be associated with its virulence
Source: Front Microbiol. 2023 Jun 15;14:1182695. doi: 10.3389/fmicb.2023.1182695 (PMC10308047; doi:10.3389/fmicb.2023.1182695)
Supplement: Supplementary file 1 [file Data_Sheet_1.zip › Supplementary Material S8.docx]

Supplementary Material S8

**The genetic variability and evolution of red-spotted grouper nervous necrosis virus quasispecies can be associated with its virulence**

**Sergio Ortega-del Campo, Luis Díaz-Martínez, Patricia Moreno, Esther García-Rosado, M. Carmen Alonso, Julia Béjar* and Ana Grande-Pérez***

*** Correspondence:** Corresponding Author: bejar@uma.es & agrande@uma.es


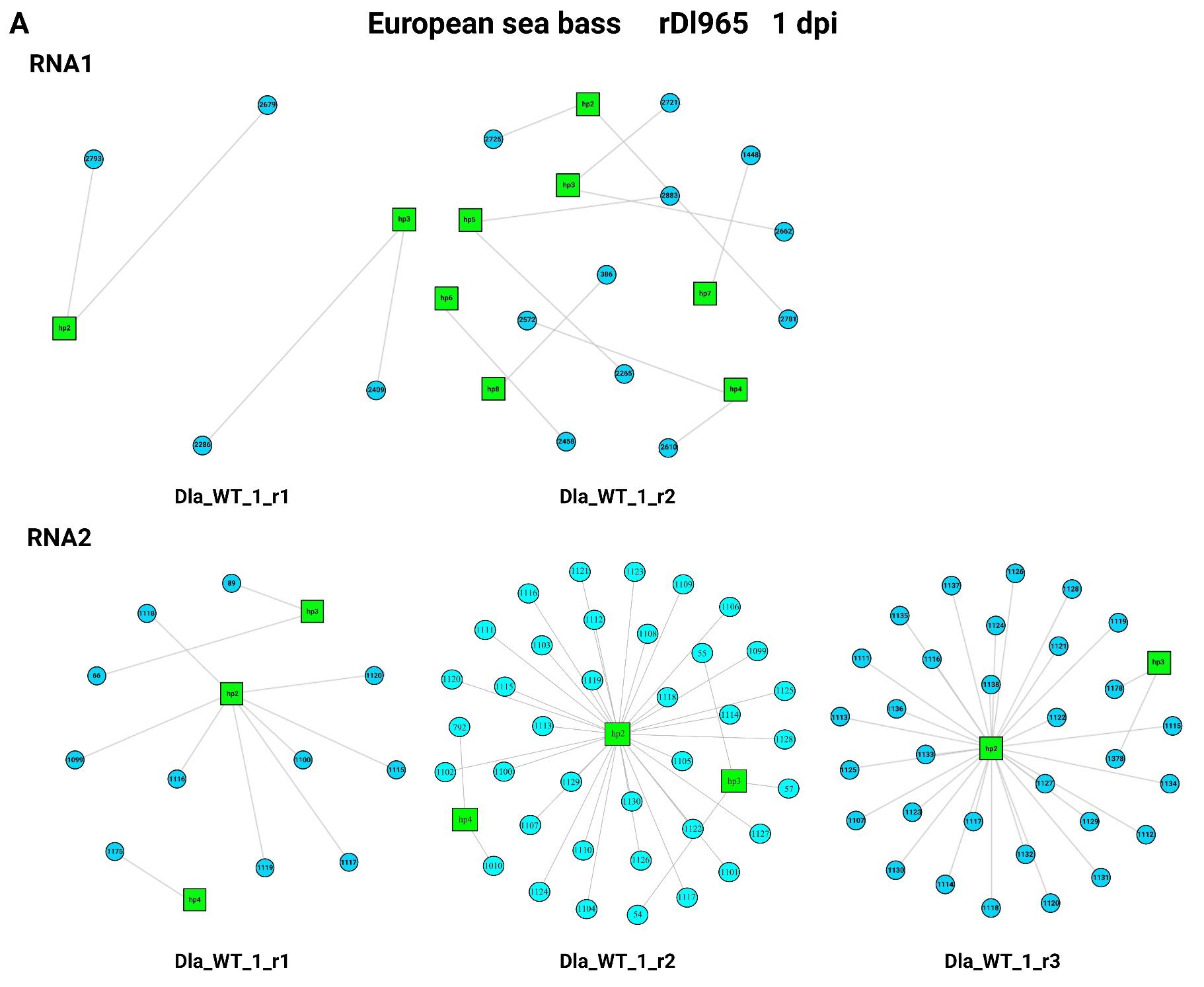


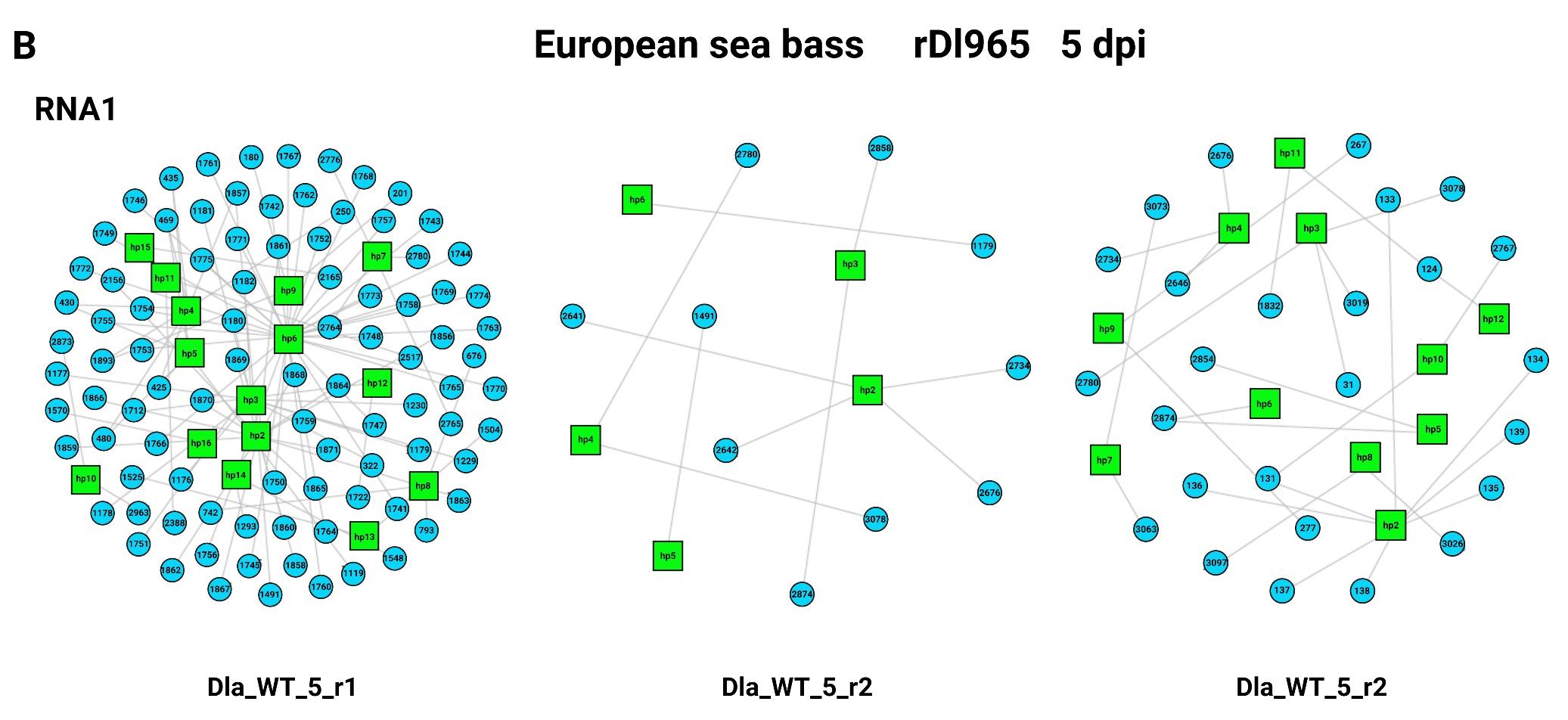


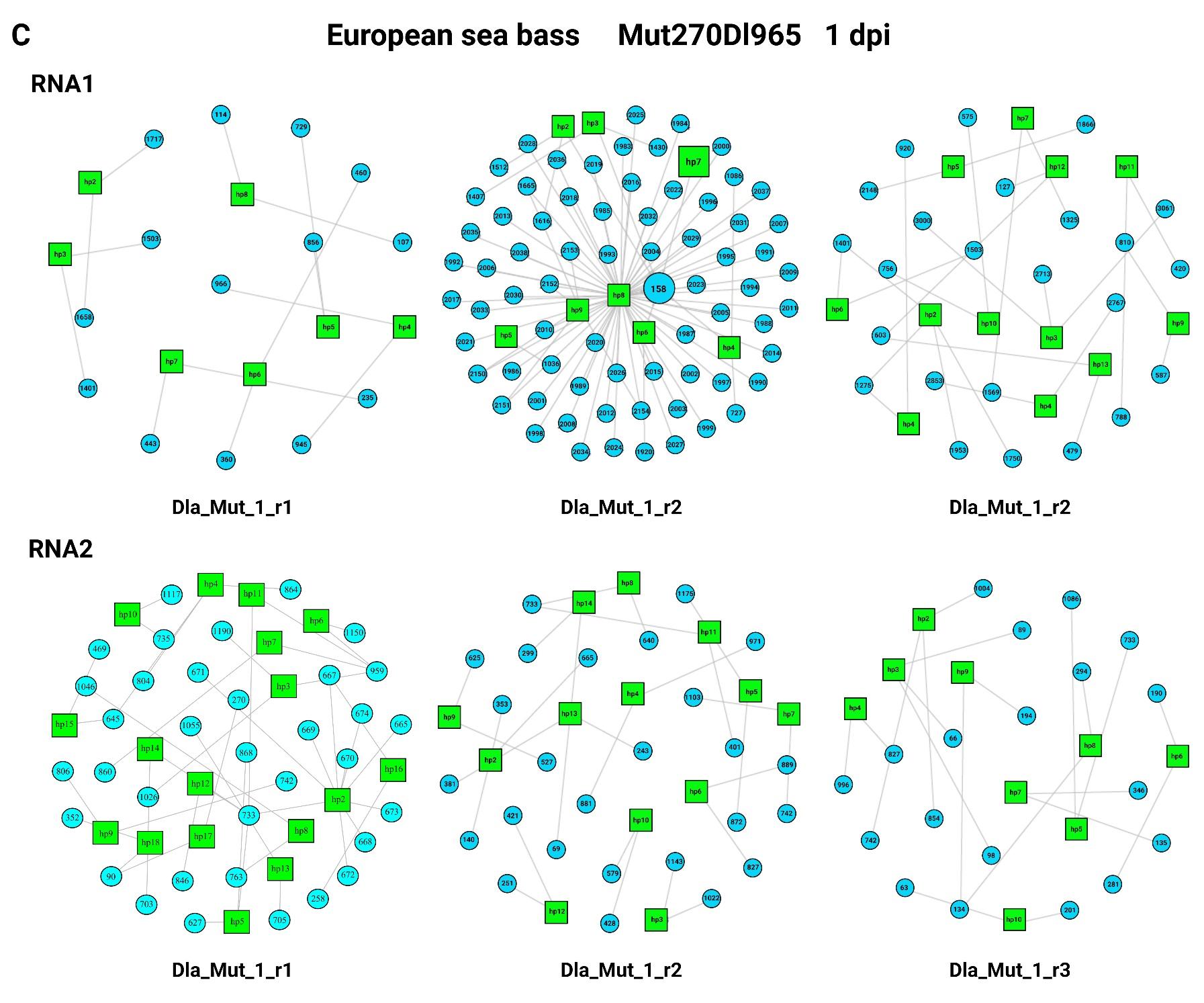


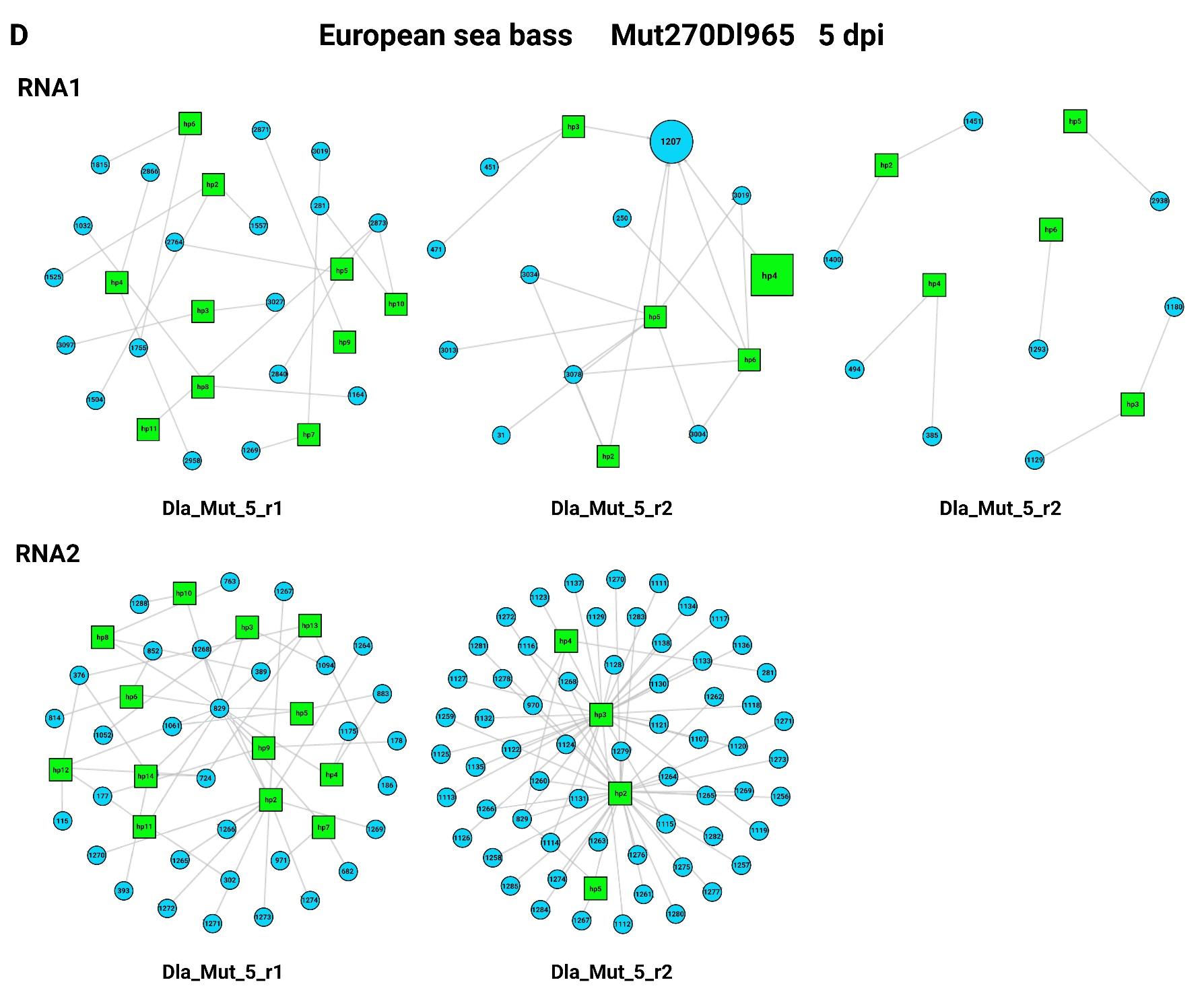


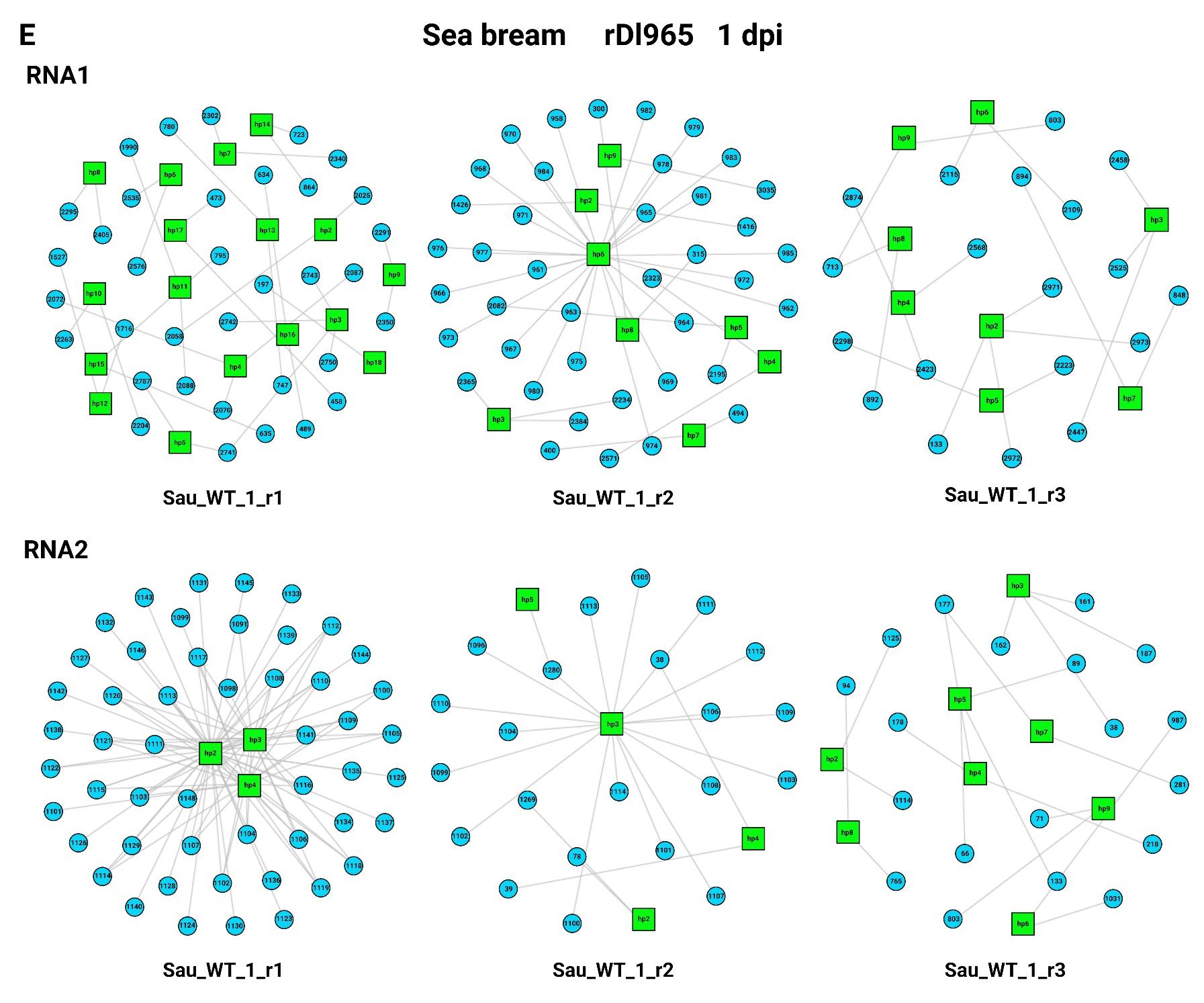


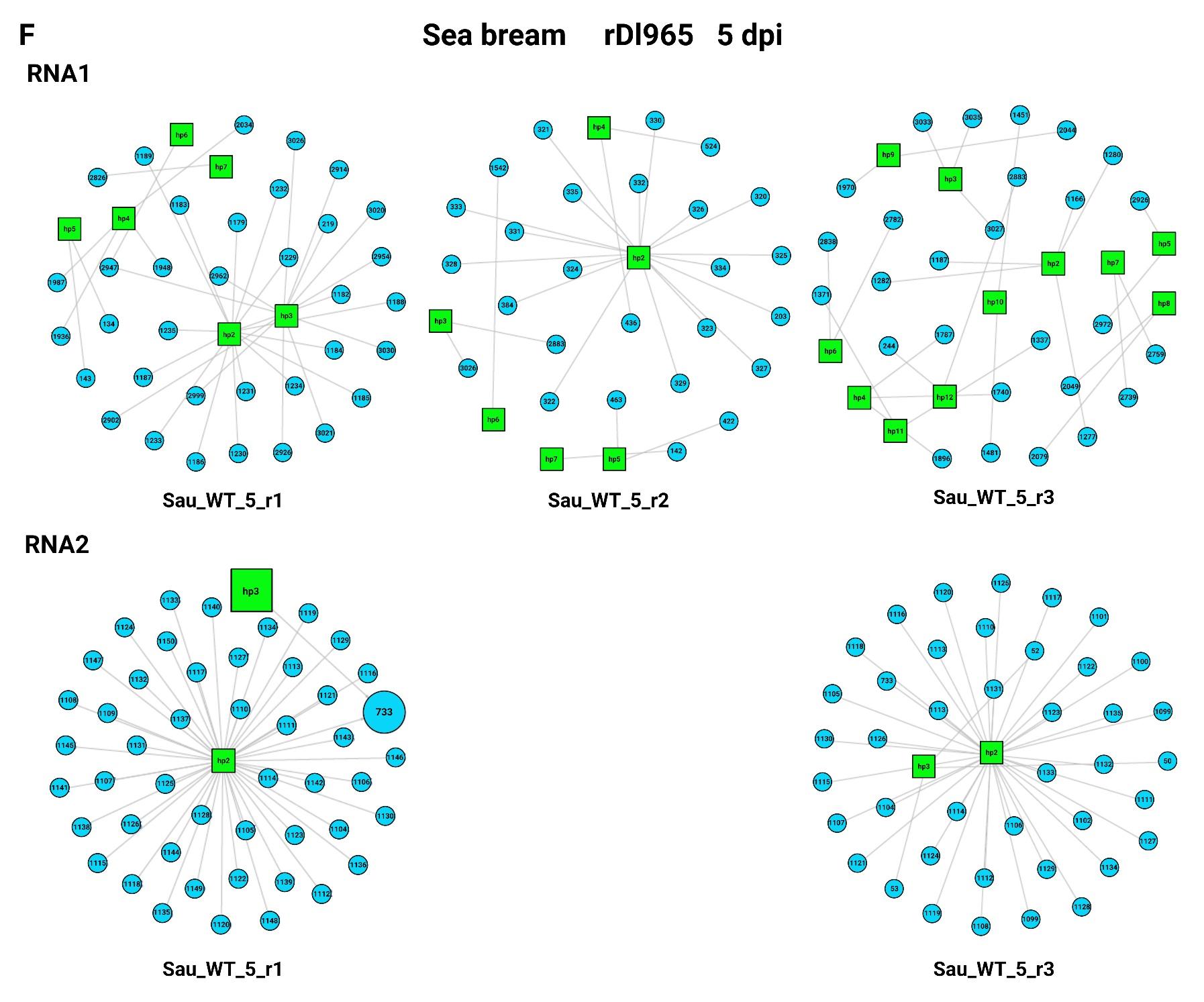


**Supplementary Figure 1.** Graphical representations of the reconstructed haplotype distributions in the red-spotted grouper nervous necrosis virus (RGNNV) quasispecies by nucleotide segment. Haplotypes are represented as green squares with the nomenclature "hp". Nucleotide changes with respect to the RGNNV consensus sequence are presented as blue circles. Each number enclosed within each circle indicates the position where the mutation was detected. The haplotypes forming the RNA2 segment networks of the Mut270Dl965 quasispecies were constructed using the original Mut270Dl965 sequence (sequence NC_008040.1 with a base change at position 830). Each panel represents the results of the samples organized according to host, virus type and day of extraction: (A) rDl965 quasispecies extracted from sea bass at 1 dpi, (B) rDl965 quasispecies extracted from sea bass at 5 dpi, (C) Mut270Dl965 quasispecies extracted from sea bass at 1 dpi, (D) Mut270Dl965 quasispecies extracted from sea bass at 5 dpi, (E) rDl965 quasispecies extracted from sea bream at 1 dpi, (F) rDl965 quasispecies extracted from sea bream at 5 dpi.
