# Supplementary material for: The genetic variability and evolution of red-spotted grouper nervous necrosis virus quasispecies can be associated with its virulence
Source: Front Microbiol. 2023 Jun 15;14:1182695. doi: 10.3389/fmicb.2023.1182695 (PMC10308047; doi:10.3389/fmicb.2023.1182695)
Supplement: Supplementary file 1 [file Data_Sheet_1.zip › Supplementary Material S9.docx]

Supplementary Material S9

**The genetic variability and evolution of red-spotted grouper nervous necrosis virus quasispecies can be associated with its virulence**

**Sergio Ortega-del Campo, Luis Díaz-Martínez, Patricia Moreno, Esther García-Rosado, M. Carmen Alonso, Julia Béjar* and Ana Grande-Pérez***

*** Correspondence:** Corresponding Author: bejar@uma.es & agrande@uma.es

**Supplementary Table 1.** Variables used by QuasiComparer workflow to cluster red-spotted grouper nervous necrosis virus (RGNNV) samples in a principal component analysis (PCA).

| **Variables** | **Definition** |
| --- | --- |
| **Transversions_rel** | Number of transversions normalized by the total amount of mapped nucleotides against the reference |
| **Transitions_rel** | Number of transitions normalized by the total amount of mapped nucleotides against the reference |
| **SNPs_rel** | Number of SNPs normalized by the total amount of mapped nucleotides against the reference |
| **InDels_rel** | Number of Indels normalized by the total amount of mapped nucleotides against the reference |
| **TsTv_ratio** | Ratio between transitions and transversions |
| **Mut_freq** | Mutation frequency: the proportion of mutant nucleotides in a population, measured as the number of mutations divided by the number of total bases. |
| **Nucleotide_div** | Nucleotide diversity: variable that measures the average number of substitutions between pairs of haplotypes in a multiple alignment. |
| **Shannon_Index** | Variable that measures heterogeneity, i.e., the proportion of different genomes present in a population. |
| **Haplotypes_tot** | Number of haplotypes assembled from the reads of a sample. |
| **Rec_freq** | Recombination frequency: number of recombinant reads divided by the total number of reads. |
| **Homo_recs** | Number of homologous recombination events. |

**Supplementary Table 2.** Statistical analysis of the variables using the QuasiComparer workflow in the PCA of the RNA1 segment of the 18 RGNNV samples.

| **Variable** | **Factor** | **p-value** |
| --- | --- | --- |
| **Haplotypes_tot** | 0.8559483 | 1.17 × 10^-5^ |
| **Shannon_Index** | 0.9331858 | 4.64 × 10^-8^ |
| **Nucleotide_div** | -0.9362829 | 3.28 × 10^-8^ |
| **Transversions_rel** | 0.7002496 | 1.75 × 10^-3^ |
| **Transitions_rel** | 0.8288452 | 3.93 × 10^-5^ |
| **SNPs_rel** | 0.8636585 | 7.93 × 10^-6^ |
| **InDels_rel** | 0.7829752 | 2.02 × 10^-4^ |
| **TsTv_ratio** | -0.5954383 | 1.17 × 10^-2^ |

**Supplementary Table 3.** Statistical analysis of the variables using the QuasiComparer workflow in the PCA of the RNA2 segment of the 18 RGNNV samples.

| **Variable** | **Factor** | **p-value** |
| --- | --- | --- |
| **Haplotypes_tot** | 0.9063246 | 5.40 × 10^-7^ |
| **Shannon_Index** | 0.7848759 | 1.90 × 10^-4^ |
| **Nucleotide_div** | -0.4901363 | 4.58 × 10^-2^ |
| **Transversions_rel** | 0.6391084 | 5.74 × 10^-3^ |
| **Transitions_rel** | 0.8678716 | 6.35 × 10^-6^ |
| **SNPs_rel** | 0.8794537 | 3.30 × 10^-6^ |
| **InDels_rel** | 0.8505459 | 1.52 × 10^-5^ |
| **TsTv_ratio** | -0.7363184 | 7.50 × 10^-4^ |
| **Mut_freq** | 0.5740532 | 1.60 × 10^-2^ |
| **Rec_freq** | -0.687536 | 2.29 × 10^-3^ |


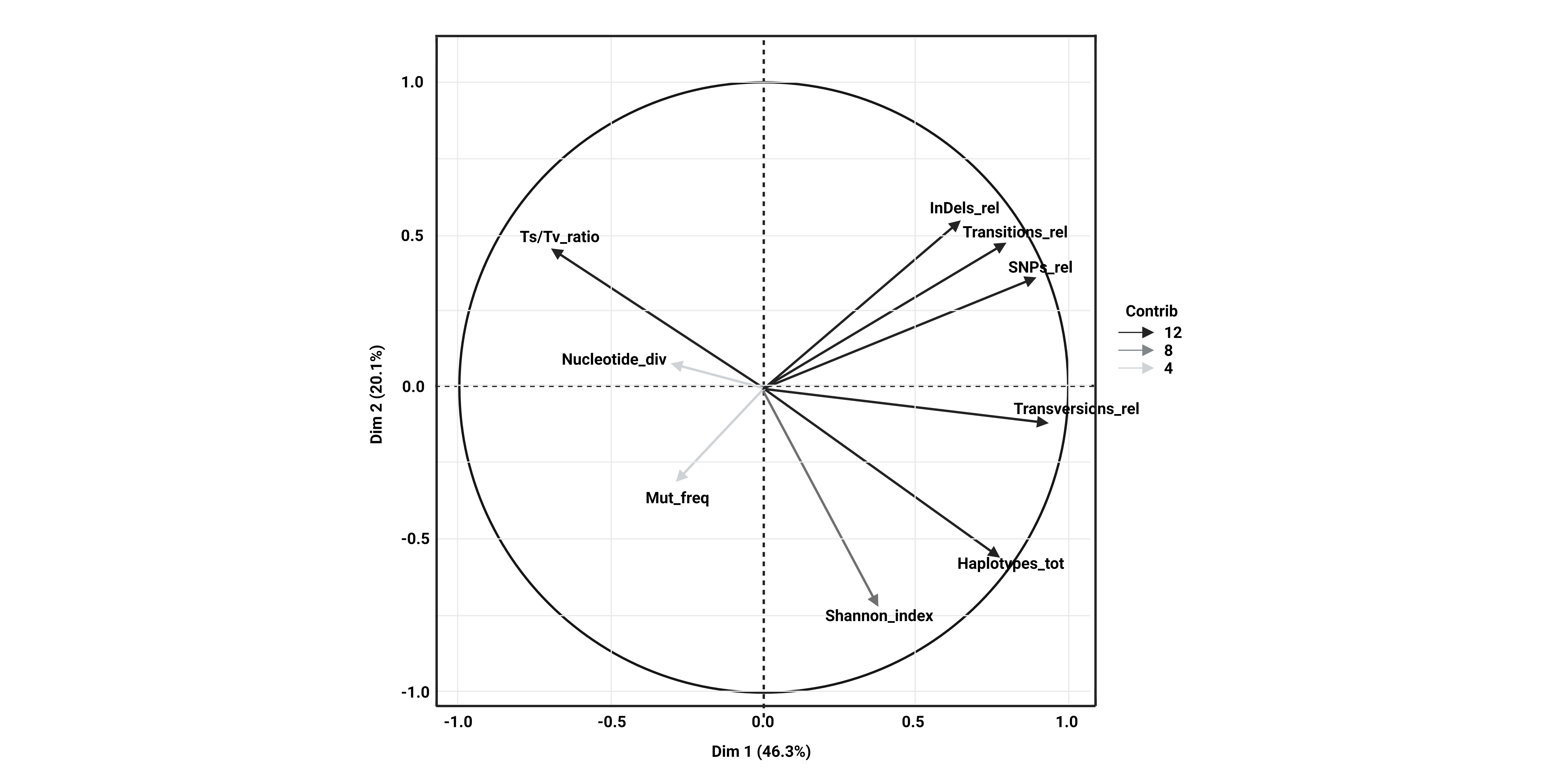

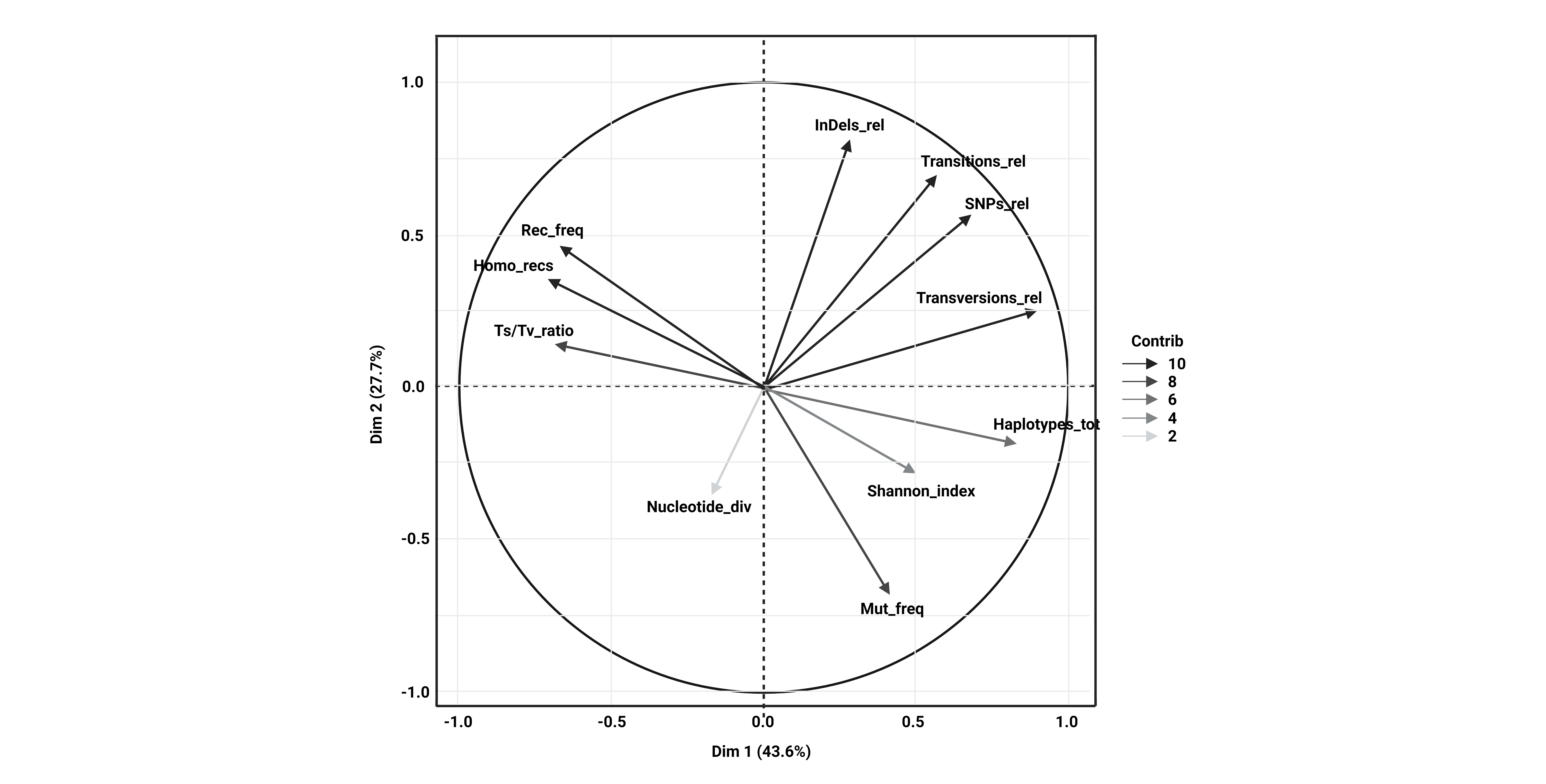


**Supplementary Figure 1.** Distribution map of the variables that define the two-dimensional space of the principal component analysis (PCA) of RNA1 (left) and RNA2 (right) of RGNNV samples. The meanings of all the variables are indicated in the Supplementary Table 1. Each variable is represented by a vector, whose direction and color intensity indicate the direction and strength with which each sample will be placed in the dimensional space of the PCA.


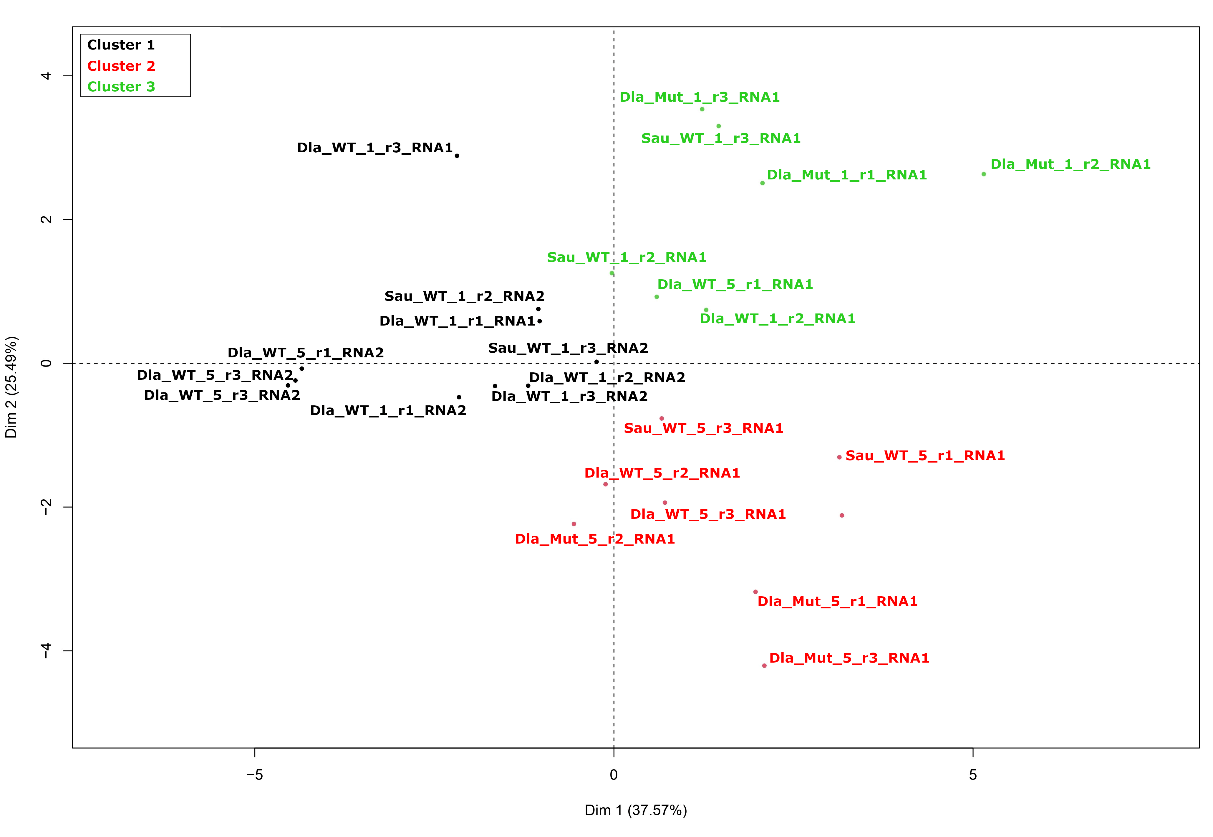

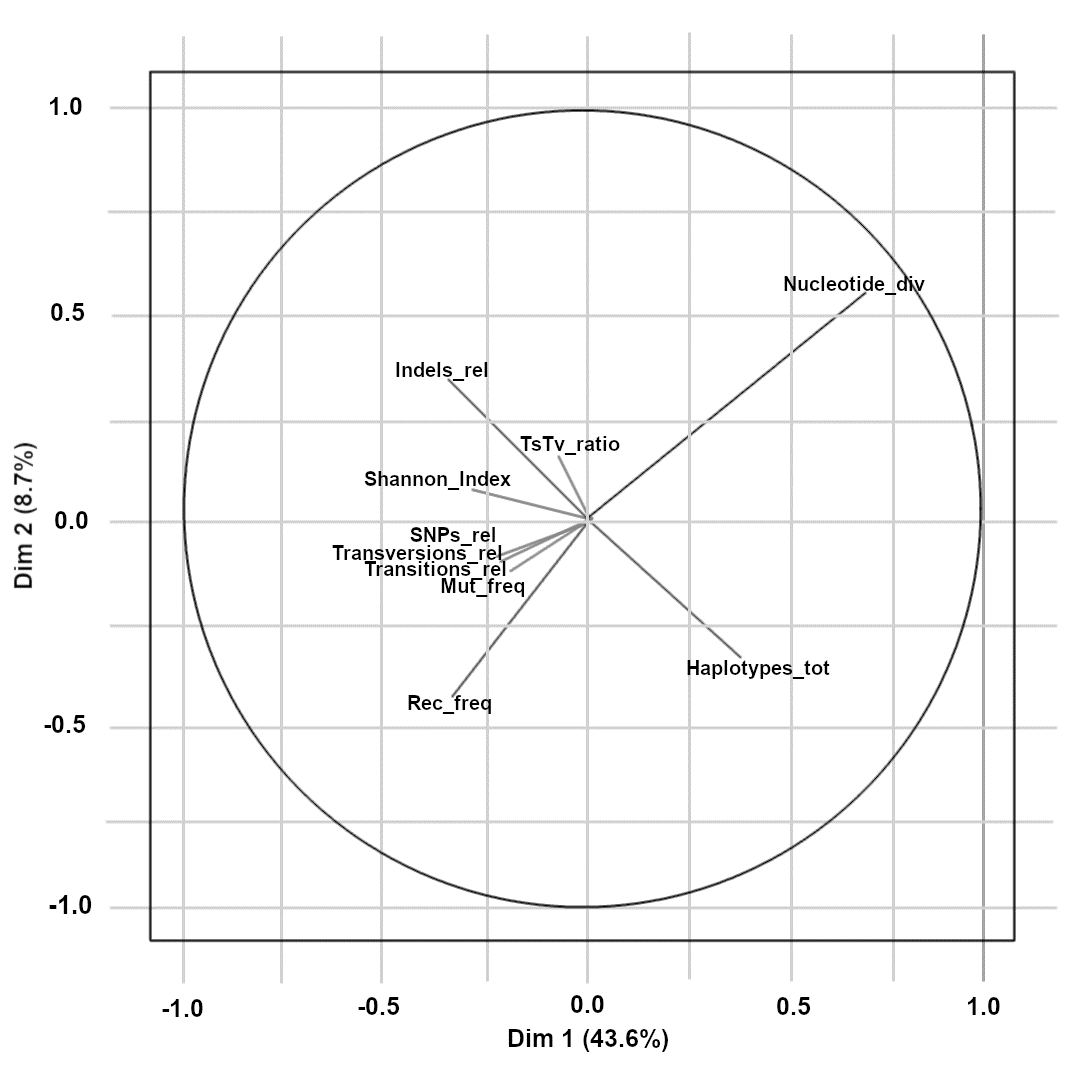


**Supplementary Figure 3.** Principal component analysis (PCA) for the RGNNV samples. PCAs were performed for RNA1 and RNA2 segments together. Left) distribution and clustering of the segments of each sample analyzed by PCA in dot plots. Right) Distribution map of the variables that define the two-dimensional space of the principal component analysis (PCA) of both segments of the viral samples.
